# A novel cell-penetrating peptide targeting calpain-cleavage of PSD-95 induced by excitotoxicity improves neurological outcome after stroke

**DOI:** 10.7150/thno.60701

**Published:** 2021-05-03

**Authors:** Sara Ayuso-Dolado, Gema M. Esteban-Ortega, Óscar G. Vidaurre, Margarita Díaz-Guerra

**Affiliations:** Instituto de Investigaciones Biomédicas “Alberto Sols”, Consejo Superior de Investigaciones Científicas-Universidad Autónoma de Madrid (CSIC-UAM), Madrid 28029, Spain.

**Keywords:** cell-penetrating peptides, excitotoxicity, neuroprotection, PSD-95, stroke

## Abstract

Postsynaptic density protein-95 (PSD-95) is a multidomain protein critical to the assembly of signaling complexes at excitatory synapses, required for neuronal survival and function. However, calpain-processing challenges PSD-95 function after overactivation of excitatory glutamate receptors (excitotoxicity) in stroke, a leading cause of death, disability and dementia in need of efficient pharmacological treatments. A promising strategy is neuroprotection of the infarct penumbra, a potentially recoverable area, by promotion of survival signaling. Interference of PSD-95 processing induced by excitotoxicity might thus be a therapeutic target for stroke and other excitotoxicity-associated pathologies.

**Methods:** The nature and stability of PSD-95 calpain-fragments was analyzed using *in vitro* assays or excitotoxic conditions induced in rat primary neuronal cultures or a mouse model of stroke. We then sequenced PSD-95 cleavage-sites and rationally designed three cell-penetrating peptides (CPPs) containing these sequences. The peptides effects on PSD-95 stability and neuronal viability were investigated in the cultured neurons, subjected to acute or chronic excitotoxicity. We also analyzed the effect of one of these peptides in the mouse model of stroke by measuring infarct size and evaluating motor coordination and balance.

**Results:** Calpain cleaves three interdomain linker regions in PSD-95 and produces stable fragments corresponding to previously described PSD-95 supramodules (PDZ1-2 and P-S-G) as well as a truncated form SH3-GK. Peptide TP95_414_, containing the cleavage site in the PDZ3-SH3 linker, is able to interfere PSD-95 downregulation and reduces neuronal death by excitotoxicity. Additionally, TP95_414_ is delivered to mice cortex and, in a severe model of permanent ischemia, significantly improves the neurological outcome after brain damage.

**Conclusions:** Interference of excitotoxicity-induced PSD-95-processing with specific CPPs constitutes a novel and promising therapeutic approach for stroke treatment.

## Introduction

Stroke is a leading cause of death, adult disability and dementia worldwide. In the ischemic type, which accounts for 85% of total stroke cases, the decline of blood perfusion produces an infarct core of irreversibly damaged tissue surrounded by an area of penumbra that is functionally impaired but metabolically active. Nevertheless, the penumbra suffers secondary neuronal death caused by glutamate overstimulation of the N-methyl-D-aspartate type of receptors (NMDARs) and consequent excitotoxicity. Calpain is an important effector of the Ca^2+^-overload derived from NMDAR-overactivation and, therefore, it is central to stroke and other acute or chronic CNS disorders also associated to excitotoxicity 1. This “modulatory protease” processes specific substrates at discrete sequences transforming their stability, location or activity 2. In the postsynaptic density (PSD), a cytoskeletal structure beneath the synaptic membrane, calpain induces an early and strong decrease of a major PSD component, the scaffold protein-95 (PSD-95) 3. In excitotoxic conditions, calpain also processes PSD-95-interacting proteins which, similarly to PSD-95, are critical for neuronal survival and function. They include the NMDAR GluN2-subunits 3, which form truncated surface receptors lacking the intracellular regions required for NMDAR association with scaffold, downstream signaling and cytoskeletal proteins. Src tyrosine kinase is similarly processed by calpain, producing a truncated enzyme which is a mediator of neuronal death in excitotoxicity and stroke 4. Likewise, the neurotrophin receptor tropomyosin-related kinase B (TrkB), also a PSD-95-interactive protein 5, is a calpain substrate 6. In stroke, cleavage of the full-length receptor (TrkB-FL) produces a shortened form very similar to TrkB-T1, a truncated isoform that lacks the tyrosine kinase domain and is considered a dominant negative receptor 7.

Therapies for ischemic stroke are largely limited to mechanical strategies or pharmacological treatment with thrombolytic drugs, although strict eligibility criteria and a narrow therapeutic window restrict treatment to a few patients 8, 9. The clinical usage of NMDAR antagonists is hampered by their lack of selectivity and inhibition of the receptor physiological activities 10. Thus, stroke neuroprotective strategies are currently exploring the interference of death signaling downstream NMDAR-overactivation or, alternatively, the prevention of aberrant downregulation of neuronal survival cascades. PSD-95 is a very promising target for both types of strategies since it plays dual roles in survival-death choices critical to disease pathology. This protein belongs to the family of Disc large homologue (DLG) multidomain polypeptides, also including synapse-associated protein (SAP)-97, PSD-93 and SAP-102. From the N-terminus, DLG proteins contain three PSD-95/Discs large/Zonula occludens-1 (PDZ) protein-interacting domains, followed by a Src homology 3 (SH3) domain and a guanylate kinase-like (GK) domain. Recent evidence suggests that PSD-95 and related proteins are functionally and structurally composed of two supramodules that together form a supertertiary protein structure 11, 12. The domains forming a particular supramodule interact with each other to assemble an integral structural unit, having functions different from those of the isolated domains or their simple addition 13. The first PSD-95 supramodule comprises the PDZ1 and PDZ2 tandem (PDZ1-2). It presents a restrained PDZ orientation that changes to a more flexible conformation, with enhanced binding affinity, upon interaction with the PDZ-ligands of the interacting proteins 14. Domains PDZ3, SH3 and GK form the second PSD-95 supramodule (P-S-G) where PDZ3 directly couples with the SH3-GK tandem in a PDZ-ligand binding-dependent manner. Thus, for example, interaction of PDZ3 with cysteine-rich PDZ-binding protein (CRIPT) can promote a conformational change and the formation of PSD-95 homotypic and heterotypic complexes 15, 16, revealing that hierarchical binding mechanisms guide the organization of the PSD-95 complexes.

At excitatory synapses, PSD-95 shapes a framework of multiple proteins 17 that organizes signal transduction and is central to glutamatergic synaptic signaling 18. A very important PSD-95 function is the assembly of a ternary complex with NMDAR-GluN2B subunits and neuronal nitric oxide synthase (nNOS), which couples NMDAR activation and Ca^2+^-influx to nNOS activation, NO production and oxidative damage 19. This complex is shaped by interaction of the GluN2B PDZ-ligand with PSD-95 PDZs (1 and 2), and a PDZ-PDZ interaction between PSD-95 PDZ2 and nNOS PDZ domain. The ternary complex is central to neuronal physiological functions, including plasticity, learning or memory, but also contributes to neuronal death in stroke. Thus, a very relevant PSD-95-targeted stroke strategy is directed to reduce nNOS-induced neurotoxicity by dissociation of GluN2B-PSD-95-nNOS complexes 20-22. This therapy is based on the use of cell-penetrating peptides (CPPs), carrier molecules having a great potential in the treatment of pathologies affecting the nervous system. In this case, the CPP contains a short Tat sequence from HIV-1, which allows attached cargoes to cross the blood-brain barrier (BBB) and plasma membrane 23, followed by a short GluN2B sequence corresponding to the PDZ-ligand 20. The resulting peptide, nerinetide (previously known as Tat-NR2B9c or NA-1), is neuroprotective in different ischemia models 20-22, and patients with iatrogenic 24 or acute ischemic 25 stroke. Interestingly, binding of CPPs containing the GluN2B PDZ-ligand to the PDZ1-2 supramodule can be greatly improved by generation of dimeric PSD-95 inhibitors such as Tat-N-dimer UCCB01-144, a neuroprotective peptide highly efficient against ischemic brain damage 26.

An important challenge to the development of PSD-95-targeted stroke therapies is processing of this protein by calpain after brain damage. It has been described that a decrease in PSD-95 levels has dramatic effects on synaptic integrity, neurotransmission and neuronal viability. Combined knockdown of PSD-95, PSD-93 and SAP-102 reduces postsynaptic α-amino-3-hydroxy-5-methyl-4-isoxazolepropionic acid receptor (AMPAR) and NMDAR-mediated synaptic transmission and PSD size 27, while an acute PSD-95 depletion induces death of hippocampal neurons 28. Additionally, a genetic deletion of the GK domain, which unpredictably inhibits PSD-95 expression, causes neurological impairment, striatal degeneration, and altered dopamine (DA)-glutamate interplay which results in increased susceptibility to excitotoxicity 29. Therefore, we need to explore a novel and complementary PSD-95-targeted stroke therapy to prevent excitotoxicity-induced protein downregulation. We have previously shown the feasibility of such strategy by interfering TrkB-FL downregulation induced by excitotoxicity 30. We developed a Tat-based CPP (TFL_457_) containing receptor sequences controlling TrkB-FL calpain-processing which allowed sustained survival signaling initiated by binding of brain-derived neurotrophic factor (BDNF) to the receptor. This neuroprotective peptide decreased the infarct size and improved the neurological outcome in a model of stroke 30. Thus, we hypothesized that maintenance of PSD-95 levels could preserve the structure and function of the network formed by this scaffold protein at the PSD and prevent neurotoxicity. To this aim, we first explored PSD-95 processing under excitotoxic conditions, analyzed the nature and stability of the resulting PSD-95 fragments and sequenced the calpain-cleavage sites. Then, we rationally designed three peptides comprising the cleavage sequences and found that one of them interfered with PSD-95 downregulation and increased neuronal viability in excitotoxicity. This neuroprotective peptide could be highly relevant for stroke therapy since it significantly improved the neurological outcome in a model of permanent stroke. These results unveil PSD-95 stabilization as an attractive and highly relevant target for stroke therapy and, perhaps, other excitotoxicity-associated diseases.

## Methods

All reagents, CPPs, biological models, software and algorithms used in the study are described in Table S1 in Supplementary Materials.

### Experimental models

All animal procedures were performed in compliance with European Union Directive 2010/63/EU and were approved by the CSIC and Comunidad de Madrid (Ref PROEX 221/14) ethics committees. The housing facilities at the Institute were approved by Comunidad de Madrid (#ES 280790000188) and comply with official regulations. All efforts were made to minimize animal suffering and reduce the number of animals sacrificed.

### Mouse model of ischemia by photothrombosis

Permanent focal ischemia was induced in the cerebral cortex of adult male Balb/cOlaHsd mice by microvascular photothrombosis as described previously 30 and detailed in Supplementary materials. The model mimics embolic or thrombotic occlusion of small arteries, frequently found in human stroke, and causes a focal brain damage with histological and MRI correlations to human patterns 31. Development of brain injury involves damage to the vascular endothelium, platelet activation and subsequent microvascular thrombotic occlusion of the irradiated region 32, selected using a stereotaxic frame. In this case, the damaged areas after irradiation in coordinates +0.2 AP, +2 ML relative to Bregma are the primary motor (hindlimb and forelimb) and somatosensory cortex. As indicated, for neuroprotection, we retro-orbitally injected a single dose (10 nmol/g or 3 nmol/g) of peptides MTMyc or MTP95_414_ (>95% purity; GenScript) 10 min after damage initiation, right after irradiation completion. These peptides are N-ter acetylated and C-ter amidated versions of TMyc or TP95_414_ (Appendix Table S1), aimed to improve plasma stability. They were prepared as 2.5 mM solutions in 0.9% NaCl and, just before injection, fresh HCO_3_NH_4_ was added to a final concentration of 44 mM to neutralize the acidity derived from trifluoracetic acid present in them. Animal sacrifice and sample preparation for immunoblot analysis or assessment of infarct volume were performed as outlined in Supplementary materials.

### Primary culture of rat cortical neurons and treatment

Primary neuronal cultures were prepared from the cerebral cortex of 18-day-old Wistar rat embryos (E18) as previously described 33, 34 with the modifications indicated in Supplementary materials. In this mixed cell cultures, growth of the glial subpopulation occurred from early times of culture and helped to maintain non-toxic glutamate levels along neuronal maturation. After 7 days *in vitro* (DIVs), further glial proliferation was inhibited by addition of cytosine ß-D-arabinofuranoside (AraC, 10 µM). Experimental treatment of mixed cultures took place after 13 DIVs. Cultures were incubated with Tat-derived CPPs (>95% purity; GenScript; 25 µM, unless otherwise indicated) before or after induction of excitotoxicity as indicated. For protease-inhibition, cultures were preincubated with the indicated compounds (10 µM CiIII, 10 µM calpeptin or 20 µM lactacystin) for 30 min (CiIII and calpeptin) or 90 min (lactacystin). Peptides and protease inhibitors remained in the medium for the duration of treatment.

### Induction of chronic or acute neuronal excitotoxicity

To induce chronic excitotoxicity, cultures were incubated for 1 to 8 h as indicated with NMDA (50 µM, unless otherwise stated) and its co-agonist glycine (10 µM), a treatment herein denoted simply as NMDA. The co-agonists induce a strong excitotoxic response in the mature neurons present in the culture but have no effect on astrocyte viability as previously described 34. For induction of acute excitotoxicity, cultures were briefly treated with NMDA (100 µM) and glycine (10 µM) for 15 to 180 min, as indicated. Cells were then washed and fed with conditioned medium without NMDAR agonists, but containing the generic antagonist DL-AP5 (200 µM). Culture proceeded to complete 24 h after damage initiation before analysis of the effects derived from acute excitotoxicity. Neuronal damage is a process irreversibly triggered after a brief exposure to NMDAR co-agonists, although widespread neuronal degeneration only develops over the following hours and does not need further NMDAR overactivation 33, 34. To analyze a potential differential effect of PSD-95-derived CPPs on acute excitotoxicity depending on the time of addition, peptides were added to cultures 1 h before NMDA treatment or right after NMDA washing, together with DL-AP5. In all cases, damage was evaluated 24 h after initiation.

### Assessment of neuronal injury in cultures

Cell viability was measured by the MTT reduction assay. At the end of treatments, MTT (0.5 mg/ml) was added to the medium and, after 2 h of incubation at 37 °C, the formazan salts formed were solubilized in DMSO and spectrophotometrically quantified at 570 nm. As primary cultures contain neurons and glial cells, we established the contribution of glia viability to the total values by exposing sister cultures to 400 μM NMDA, 10 μM glycine for 24 h before MTT assay. These conditions induce nearly complete death of mature neurons and no glial damage 34. After subtracting this absorbance value, contributed by quiescent glial cells, we obtained the viability of the neuronal subpopulation in each sample. All experiments included sample triplicates for each treatment, and multiple independent experiments were carried out as detailed in the figure legends.

### Peptide visualization in brain cortex

Mice were retro-orbitally injected with vehicle (saline), biotin-labeled TMyc (Bio-TMyc) or TP95_414_ (Bio- TP95_414_) (Table S1 in Supplementary Materials), used at 3 nmol/g. Thirty minutes after peptide administration, animals were deeply anesthetized, intracardially perfused and brains post-fixed and cryoprotected as indicated for immunohistochemistry (Supplementary Materials). Coronal frozen sections (30 μm thick) were then incubated in blocking solution (10% goat serum, 0.5% Triton X-100 in PBS) for 4 h at room temperature. After washing, sections were incubated for 1 h with Fluorescein Streptavidin (1 µg/ml) and DAPI (5 µg/ml). Sections were mounted and dried on slides, and cover slipped with DPX. Images were obtained with a 63× objective, correspond to single sections and were processed for presentation with ImageJ (NIH Image).

### *In vitro* calpain proteolysis

Protein extracts were prepared from adult brain cortex, neurons or HEK293T cells by using radioimmunoprecipitation assay (RIPA) buffer (50 mM Tris-HCl pH 8, 150 mM NaCl, 1% sodium deoxycholate, 1% NP-40, 1 mM DTT and 0.1% SDS for cultures or 1% SDS for tissue), containing 1 mM phenylmethylsulfonyl fluoride, 10 μg/ml pepstatine, 10 μg/ml aprotinin, 1 mM EDTA and 1 mM EGTA, and incubated for 30 minutes at 4°C. Protein concentration was determined with BCA Protein Assay Kit (Thermo Fisher). Processing was initiated by addition of pure calpain I (5-100 U/ml) or II (50-200 U/ml) to diluted extracts supplemented with 5 mM DTT and 2.5 mM CaCl_2_, and incubation proceeded for 30 minutes at 37 °C. When indicated, Tat-derived CPPs (0.125, 0.250 and 0.500 µM) were added to the reaction immediately before protease treatment.

### *In silico* prediction of calpain putative cleavage sites and IDRs

Calpain cleavage in PSD-95 was predicted using several algorithms provided by Calpain Modulatory Proteolysis Database (CaMPDB) and GPS-Calpain Cleavage Detector (GPS-CCD) and SitePrediction. IDRs were analyzed using the online GeneSilico Metadisorder service. Residues whose disorder probability is over 0.5 are considered as disordered. Details of these tools are in Table S1.

### Edman N-terminal sequencing of calpain cleavage sites

Total lysates from HEK293T cells expressing pNice-PSD-95-YFP fusion protein (5 mg) were digested with 100 U/ml of purified calpain I, as before. The C-terminal fragments generated after *in vitro* proteolysis were immunoprecipitated using a rabbit polyclonal anti-GFP antibody immobilized on Protein A-Sepharose, resolved in SDS-PAGE, transferred to PVDF membranes and stained with Coomasie Blue. The bands of interest were cut, washed twice and kept at 4 °C until Edman degradation analysis. Sample duplicates were run in parallel and analyzed by immunoblot with the GFP antibody in order to ensure that electrophoretic mobility of purified bands matched that previously detected in HEK293T digestions. Edman N-terminal sequencing was performed by the Proteomic Facility of the “Centro de Investigaciones Biológicas” (CSIC, Madrid, Spain). Six rounds of protein degradation followed by HLPC identification of excised amino acids revealed the sequences of interest.

### Measurement of infarct volume

Rostral and caudal images of TTC-stained coronal slices were analyzed using ImageJ software by an observer blinded to experimental groups. After image calibration, delineated areas of ipsilateral and contralateral hemispheres, and the infarcted region (unstained area) were measured. Considering slices thickness, the corresponding volumes were calculated and corrected for edema's effect, estimated by comparing total volumes of hemispheres. The corrected infarct volumes were expressed as percentage relative to the contralateral hemisphere, to correct for normal size differences between different animals. For each animal, the mean of results obtained for rostral and caudal sides was calculated.

### Beam walking test

Motor coordination and balance were evaluated in mice right before and 24 h after the ischemic insult by measuring the number of contralateral hind paw slips in the beam walk apparatus. Mice had to walk through a narrow beam (1 m × 1 cm × 1 cm) placed 50 cm above the tabletop, going from an aversive stimulus (60-watt light bulb) to a black goal box with nesting material. Slips taking place in a previously selected central beam-segment (50 cm long) were counted. Before damage induction, mice were allowed to cross the beam once, to get acquainted with the test, which they repeated 24 h after photothrombosis, immediately before sacrifice.

### Quantification and statistical analysis

All data were expressed as mean ± standard error of the mean (SEM) of at least three independent experiments. The details of the number of experiments done, the precise sample size and the specific statistical test applied for each case can be found in the figure legends. For *in vivo* studies, animals were randomly allocated to the experimental groups and the researchers doing the experiments were blind respect to treatment. We pre-established an exclusion criterion for values more than three standard deviations outside the mean, considered as outliers. For the *in vitro* studies, cell samples in each independent experiment were sister primary cultures grown in multiwell plates, obtained from the same cell suspension. Treatments were assigned in a random way and biological replicates (n=3) were included for most experiments as indicated. Multiple independent experiments were then carried out as detailed in the figure legends. Considering the number of groups, the data distribution and the homogeneity of variances, unpaired Student's t-test, one-way ANOVA, two-way ANOVA or a generalized linear model were used. In all cases, when significant differences were found, a multiple comparison test (LSD *post-hoc* test) was used. Data were represented as per cent of controls or maximum values as indicated. A *p* value smaller than 0.05 was considered statistically significant (*p<0.05, **p<0.01, ***p<0.001). Statistical analysis was performed with the Statistical Package for Social Science (SPSS, v.18, IBM).

## Results

### Specific PSD-95 downregulation induced by *in vitro* excitotoxicity produces a consistent number of stable fragments

**Figure 1:**
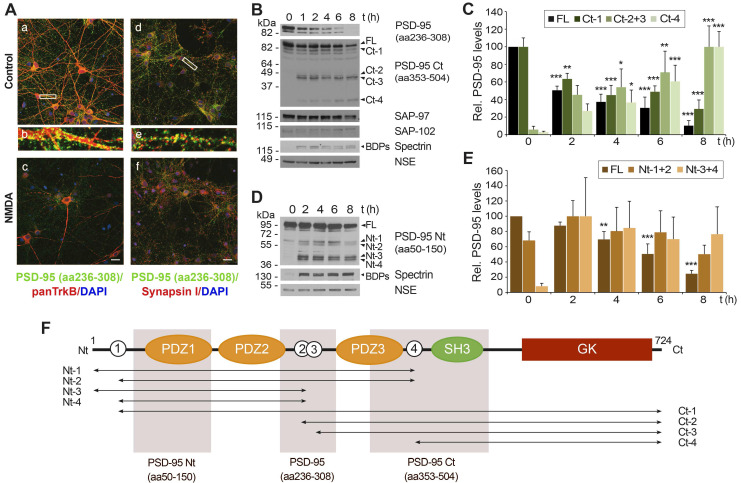
** Specific PSD-95 downregulation induced by *in vitro* excitotoxicity produces a consistent number of stable fragments.** (A) Analysis of PSD-95 downregulation by immunofluorescence. Primary cortical cultures of 14 days *in vitro* (DIVs) were incubated for 1 h with NMDA (100 µM) and glycine (10 µM), herein denoted simply as “NMDA”, or left untreated. Double immunofluorescence was performed with antibodies recognizing aa 236 to 308 in PSD-95 (green) and a panTrkB antibody directed to an extracellular domain shared by full-length and truncated TrkB forms or a synapsin I antibody (both in red). Nuclei were stained with DAPI (blue). Enlarged images show details of areas marked by rectangles in control samples. Images are representative and correspond to single sections. Scale bar, 10 µm. (B) Time-course and specificity of PSD-95 processing to produce C-terminal fragments. Immunoblot analysis of cultures treated with NMDA (1-8 h) or left untreated using two PSD-95 antibodies recognizing respectively aa 236-308 or 353-504 (herein denoted as PSD-95 Ct) or antibodies for SAP-97 and SAP-102. Formation of characteristic breakdown products (BDPs) due to spectrin cleavage and stable NSE levels are also shown. For PSD-95, arrows point to the full-length (FL) protein and main C-terminal fragments (Ct-1 to Ct-4) produced. (C) Quantitation of PSD-95 downregulation and formation of relatively stable Ct fragments. Protein levels were normalized to NSE and expressed relative to the maximum value obtained for each polypeptide (untreated cultures for FL and Ct-1 or 8 h-treatment for other Ct-fragments), arbitrarily given a 100% value. Means ± SEM were represented (n = 3-4) and analysis was performed by a generalized linear model followed by a LSD *post-hoc* test. *p < 0.05, **p < 0.01 and ***p < 0.001. (D) Time-course of production of N-terminal fragments. Analysis of cultures treated with NMDA (2-8 h) as before using antibodies recognizing PSD-95 aa 50-150 (herein denoted as PSD-95 Nt). Arrows point to the FL protein and main N-terminal fragments (Nt-1 to Nt-4). (E) Quantitation of PSD-95 downregulation and formation of relatively stable Nt fragments. Protein levels were normalized and quantified as before. Means ± SEM were represented (n = 3-4). (F) Diagram of PSD-95 domains and sequences recognized by antibodies. From the N-terminal (Nt) to the C-terminal (Ct), we observe three PDZ (PDZ1 to 3; orange) domains, a Src homology 3 (SH3; green) domain and a Guanylate kinase-like (GK; dark red) domain. Also indicated is the approximate location of at least four N-terminal and four C-terminal calpain-fragments (labeled respectively Nt-1 to Nt-4 and Ct-1 to Ct-4), inferred from immunoblots. Four predicted cleavage sites are represented as white circles.

To design a neuroprotective strategy based on prevention of PSD-95 calpain-processing induced by NMDAR-overactivation, we first analyzed the nature and stability of the fragments resulting from cleavage. Mature cortical neurons were treated with high concentrations of the NMDAR co-agonists, NMDA and glycine, herein denoted simply as “NMDA”, and analyzed using an antibody that recognizes PSD-95 residues 236 to 308 by immunofluorescence (Figure 1A) and immunoblot (Figure 1B, upper panel). Double immunofluorescence was performed in combination with panTrk, antibody recognizing all different TrkB isoforms and predominantly labeling the soma and dendrites of untreated neurons, or an antibody specific for the presynaptic protein synapsin I. As expected, PSD-95 partially colocalized with interacting-protein TrkB 5 while it occupied nearby positions opposite to synapsin I in the membrane of control neurons (Figure 1A, respectively panels a and d; see also panels b and e for enlargements). After a brief NMDA stimulation (1 h), panTrkB still labeled neurons which presented characteristic morphological changes preceding excitotoxic death such as dendritic focal swelling and formation of varicosities 35. Levels of PSD-95 were strongly reduced by NMDA whereas partial colocalization with TrkB was lost and proximity to synapsin I decreased. Immunoblot analysis with this antibody confirmed early PSD-95 downregulation and a further decrease by extended chronic treatment (Figure 1B, upper panel) as described 3. Regulation of PSD-95 by NMDAR-overactivation was specific and did not occur for other DLG proteins also interacting with NMDARs, such as SAP-97 or SAP-102. Other neuronal proteins such as neuron-specific enolase (NSE) were not affected by NMDA and, thus, used as loading control. Meanwhile, activation of calpain in excitotoxicity was confirmed by the accumulation of characteristic spectrin breakdown products (BDPs; 150 and 145 kDa).

Calpain-processing of PSD-95-interacting proteins TrkB 6, 36 or NMDAR-GluN2 subunits 3 induced by excitotoxicity or ischemia produces relatively stable cleavage fragments. However, we could not detect fragments with PSD-95 (aa236-308) antibody. Thus, we repeated the analysis with additional antibodies recognizing different PSD-95 sequences (summarized in Figure 1F). An antibody directed to the C-terminal half of PSD-95 (aa353-504, denoted as PSD-95 Ct) showed that the decrease of the full-length (FL) protein and a big fragment already present in basal conditions (Ct-1; Figure 1B) was accompanied by progressive formation of smaller C-terminal fragments (Ct-2 to Ct-4), predicting at least four calpain-targets approximately located in interdomain PSD-95 sequences (white circles numbered 1 to 4 in Figure 1F). Quantitation of these results demonstrated that NMDA induced a strong reduction of normalized levels of PSD-95 FL and Ct-1 (Figure 1C) which correlated with a reciprocal increase of the other fragments (Ct-2 and Ct-3, having similar size and thus pooled together, and Ct-4) and was concomitant with calpain activation. These PSD-95 fragments accumulated with the time of treatment and reached maximum levels at 8 h. This result suggested a substrate-product relationship between PSD-95 and the Ct-fragments. To complete this analysis, we performed similar experiments with a PSD-95 antibody directed to the N-terminus (aa50-150, denoted as PSD-95 Nt; Figure 1D) and quantified the results (Figure 1E). The size of the main fragments obtained (Nt-1 to Nt-4) was compatible with them being complementary to the Ct-fragments previously shown. The higher levels of Nt-3 and Nt-4 compared to Nt-1 and Nt-2 suggested than calpain-cleavage at sites 2 or 3 (Figure 1F, white circles) was more efficient than processing at site 4. Altogether, above results demonstrate that PSD-95 downregulation induced by excitotoxicity is specific and produces a limited set of relatively stable fragments that probably correspond to at least 4 different calpain-cleavage sequences.

### Both acute and chronic NMDAR-overactivation induce PSD-95 downregulation dependent on Ca^2+^-influx and calpain activation

**Figure 2:**
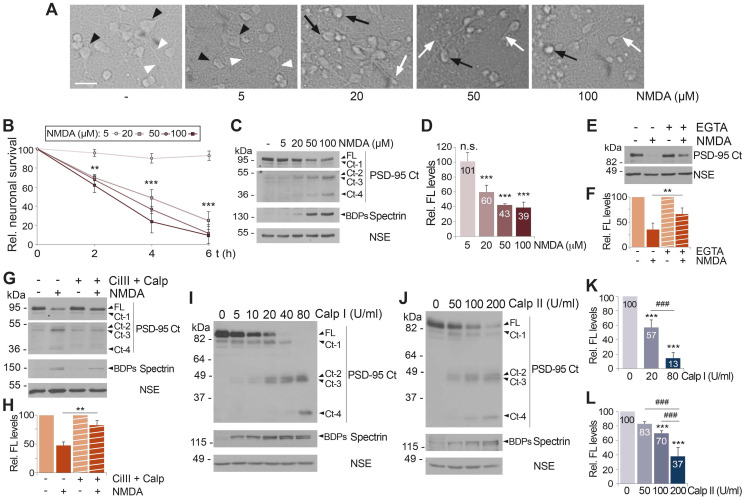
** Acute and chronic NMDAR-overactivation induce PSD-95 downregulation dependent on Ca^2+^-influx and calpain activation.** (A) Effect of chronic NMDAR-overactivation in neuronal morphology. Primary cultures incubated for 4 h with different concentrations of NMDA were compared to untreated cells. Cells with neuronal morphology (black arrowheads) and neurites (white arrowheads) characteristic of this *in vitro* maturation time are shown in untreated cultures or those incubated with 5 µM NMDA. Hallmarks of the excitotoxic process such as soma vacuolization (black arrows) or neurite varicosities (white arrows) can be observed after treatment with NMDA concentrations ≥ 20 µM. Scale bar, 50 μm. (B) Effect of chronic NMDAR-overactivation on neuronal survival. Cultures were treated as before for 2, 4 or 6 h. Specific neuronal viability was established and expressed relative to values in the untreated cultures, arbitrarily assigned a 100% value. Means ± SEM were represented (n = 7) and analysis was performed by a generalized linear model followed by a LSD *post-hoc* test. **p < 0.01, ***p < 0.001. (C) Dose-dependent effect of chronic NMDA treatment on PSD-95 levels. Cultures treated as before for 4 h were compared to untreated cells. Levels of PSD-95 (FL or Ct-1 to Ct-4) were established with PSD-95 Ct. Two different exposures are presented to facilitate visualization of C-terminal fragments. (D) Quantitation of PSD-95 downregulation induced by chronic NMDAR-overactivation. PSD-95 FL levels were normalized to NSE and expressed relative to values obtained in cultures without NMDA. Means ± SEM (n = 3-5) were represented and statistical analysis was performed by a generalized linear model followed by a LSD *post-hoc* test. ***p < 0.001, n.s. = non-significant. (E) Dependence on calcium influx. Cultures were preincubated for 2 h with the Ca^2+^ chelator EGTA (2 mM) before acute NMDA treatment (100 µM, 1 h). To prevent possible toxic effects of long treatments with chelator, EGTA and NMDA were then removed and culture proceeded for 23 additional hours in conditioned media containing the NMDAR-antagonist DL-AP5 (200 µM). (F) Quantitation of the dependence on Ca^2+^-influx of excitotoxic PSD-95 processing. Protein FL levels obtained in cultures treated as above were normalized to NSE and expressed relative to values obtained in cells without NMDA. Means ± SEM (n = 5) were represented and the statistical analysis was performed by ANOVA test followed by a LSD *post-hoc* test. The comparison of cells treated with NMDA, with or without the Ca^2+^ chelator, is shown. **p < 0.01. (G) PSD-95 processing by a calpain-dependent mechanism during excitotoxicity. Cultures were preincubated 30 min with calpain inhibitors III (CiIII, 10 µM) and calpeptin (10 µM) before NMDA treatment for 4 h. (H) Quantitation of the dependence of excitotoxic PSD-95 processing on calpain activity. PSD-95 FL levels obtained in cultures treated as described were normalized to NSE and expressed relative to values obtained in cells without NMDA. Means ± SEM (n = 5) were represented and analyzed as above. The comparison of cells treated with NMDA, with or without protease inhibitors, is shown. **p < 0.01. (I and J) PSD-95 susceptibility to *in vitro* calpain-processing. Protein extracts prepared from primary cultures were incubated with pure calpain I (5-80 U/ml; panel I) or II (50-200 U/ml; panel J) for 30 min. Calpain I and II activities were confirmed by analysis of spectrin BDPs. (K and L) Quantitation of PSD-95 susceptibility to *in vitro* calpain-processing. PSD-95 FL levels were expressed relative to values obtained in the untreated protein extracts. Means ± SEM (n = 4) were represented and analyzed as above. Comparisons with the untreated extracts (***p < 0.001) or between two different calpain concentrations (^###^*p* < 0.001) are shown.

To deepen our understanding of PSD-95 downregulation, we first defined whether the mechanism induced by NMDA was associated to excitotoxic NMDAR stimulation or could be also observed with low agonist concentrations. Neuronal morphology characteristic of excitotoxicity 35 was only observed for NMDA concentrations higher than 5 µM (Figure 2A) and, accordingly, similar concentrations were required to decrease neuronal viability (Figure 2B), in agreement to a previously described nonlinear response to NMDA 3, 37. A similar dose-response was observed for PSD-95 levels (Figure 2C-D). This dose-response was similar to that of PSD-95-interacting protein TrkB-FL 6, although PSD-95 appeared to be more sensitive to low NMDA concentrations (Figure S1). Previous results suggested that PSD-95 downregulation was associated with excitotoxic stimulation of NMDARs and concurrent with neuronal death. Accordingly, PSD-95 processing was also induced by glutamate and, similarly to NMDA, prevented by competitive (DL-AP5) or non-competitive (memantine and ketamine) NMDAR antagonists (Figure S2A). Ifenprodil, a selective inhibitor of NMDAR-GluN2B subunits 38, blocked the PSD-95 decrease similarly to DL-AP5 (Figure S2B). Since cultured neurons expressed both GluN2A and GluN2B (Figure S2B), these results suggest that PSD-95 downregulation requires overactivation of NMDARs containing GluN2B subunits. Finally, acute stimulation of neurons with NMDA for 15 minutes was sufficient to induce an irreversible reduction in the PSD-95 levels detected 24 h later that could not be blocked by antagonists (Figure S2C). This result agrees with excitotoxicity being an irreversible process that, after a critical period of time, cannot be inhibited or reverted by NMDAR antagonists 39.

Activation of NMDARs leads to Ca^2+^-influx due to a high receptor permeability for this ion. Thus, we analyzed if PSD-95 downregulation was dependent on Ca^2+^-entry by comparing cultures preincubated or not with Ca^2+^ chelator EGTA before the induction of excitotoxicity (Figure 2E-F). Buffering of extracellular Ca^2+^ partially prevented the decrease of PSD-95 induced by NMDA, suggesting that PSD-95 downregulation is triggered by calcium entry due to NMDAR-overstimulation. Next, we confirmed the participation of calpain, activated by Ca^2+^-influx, in PSD-95 cleavage (Figure 2G-H). Cultures preincubated with calpain inhibitors, calpain inhibitor III (CiIII) and calpeptin (Calp), before NMDA treatment showed limited calpain blockade compared to cells treated only with NMDA, paralleled by partial recovery of PSD-95 FL and decrease of Ct-fragments. Globally, above results suggest that PSD-95 downregulation is triggered by both acute and chronic overactivation of NMDARs containing GluN2B subunits, requires Ca^2+^-influx and is due to calpain activation. Nevertheless, these results did not exclude the participation of additional mechanisms. Regulation of PSD-95 by the ubiquitin-proteasome pathway in response to physiological NMDAR activation is important for synaptic plasticity 40. Thus, this proteolytic system might be also involved in PSD-95 degradation after NMDAR-overactivation. However, culture preincubation with the specific proteasome inhibitor lactacystin before excitotoxicity did not prevent PSD-95 downregulation although it increased levels of proteasome substrate p53 (Figure S3A). We also considered a potential participation of transcriptional repression since this type of control is important in the excitotoxic regulation of GluN1 subunits 41 and Kidins220/ARMS 42, a protein that similarly to PSD-95 interacts with both NMDARs and TrkB receptors. However, analysis of PSD-95 mRNA levels in neurons incubated with NMDA showed no significant differences to those obtained in the untreated cells (Figure S3B).

Previous results strongly suggested that PSD-95 was a novel calpain-substrate. Thus, we analyzed the susceptibility of this protein to *in vitro* processing by different concentrations of purified calpain I (Figure 2I) or calpain II (Figure 2J). The emergence of spectrin BDPs confirmed progressive calpain activation and parallel processing of PSD-95 FL, rendering Ct-fragments of mobility similar to that previously found in the *in vitro* model of excitotoxicity (Figure 1B). Quantitation of these experiments demonstrated a dose-response in PSD-95 processing both for calpain I (Figure 2K) and calpain II (Figure 2L). Collectively, these results demonstrate that PSD-95 is a calpain-substrate and that activation of this protease as a consequence of Ca^2+^-influx induced by acute or chronic NMDAR-overactivation is a major mechanism of PSD-95 downregulation.

### Permanent focal brain ischemia induces early PSD-95 downregulation through mechanisms similar to those found *in vitro*

**Figure 3:**
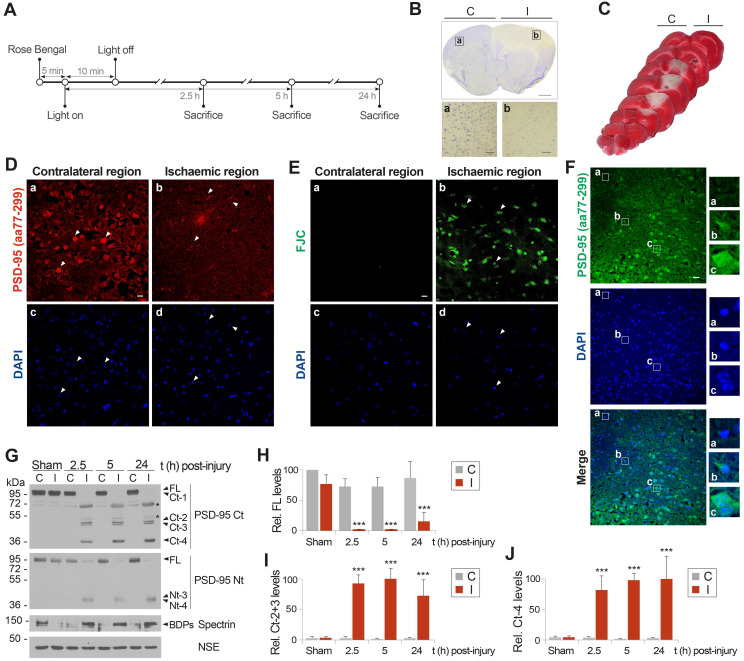
** Permanent focal brain ischemia induces early PSD-95 downregulation through mechanisms similar to those found *in vitro.*** (A) Experimental design to characterize PSD-95 regulation in ischemia. After Rose Bengal i.v. injection, permanent vessel occlusion and focal brain damage was induced by cold-light irradiation. Animals were sacrificed as different times after damage. (B and C) Assessment of ischemic damage by photothrombosis (24 h). Nissl staining of coronal sections (B) revealed a hypochromatic area indicative of neuronal injury in the ipsilateral neocortex (I), compared with equivalent regions of the contralateral hemisphere (C). Scale bar: 1 mm. Details of representative regions (a and b) are also presented. Scale bar: 20 µm. TTC staining of coronal slices (C). (D and E) Association of PSD-95 downregulation and neurodegeneration after ischemia. The ischemic region and the equivalent area of contralateral hemisphere were compared in brain coronal sections of animals sacrificed 5 h after damage. Cells expressing PSD-95 (red) and their corresponding nuclei (blue) are denoted by arrowheads (D). Neurodegeneration was analyzed in contiguous sections by Fluoro-Jade C (FJC) (green) and DAPI (blue) staining. Arrowheads denote cell bodies of degenerating neurons and their condensed nuclei (E). Representative images correspond to single sections. Scale bar: 10 µm. (F) Detailed analysis of PSD-95 expression (green) and nuclei condensation (blue) in the region separating the ischemic and non-ischemic areas. Scale bar: 20 µm. Enlarged images of representative cells (a to c) corresponding to different levels of nuclear damage are also presented. (G) PSD-95 analysis in cortical infarcted regions (I) and equivalent areas of the contralateral hemisphere (C) in mice sacrificed at the indicated times. (H, I and J) Quantification of normalized levels of PSD-95 FL (H) or fragments Ct-2 + Ct-3 (I) or Ct-4 (J) in contralateral and infarcted regions. Data were expressed as a percentage of the value obtained in the contralateral area of sham-operated mice for PSD-95 FL (H) or the maximum levels of Ct-fragments (I and J), arbitrarily given a 100% value. Means ± SEM were represented (n = 3) and analysis was performed by a generalized linear model followed by a LSD *post-hoc* test. ***p < 0.001.

We previously obtained preliminary results showing a decrease of PSD-95 in the infarcted brain of rats subjected to transient middle cerebral artery occlusion (MCAO) 3. To develop a neuroprotective strategy based on prevention of PSD-95 calpain-processing, we needed to further investigate *in vivo* cleavage of this protein induced by NMDAR-overactivation. We used a mouse model of focal permanent ischemia induced by microvascular photothrombosis 32 (summarized in Figure 3A). This model produces infarcts with similar histological and magnetic resonance imaging patterns to stroke patients 31, frequently caused by embolic or thrombotic occlusion of small arteries. Cortical infarcts in motor and somatosensory areas of the ipsilateral hemisphere (I), established as hypochromatic areas after Nissl staining (Figure 3B) or TTC unstained regions (Figure 3C), were obtained in animals sacrificed at different times after damage induction and compared to equivalent regions of the contralateral hemisphere (C). Five hours after damage induction, immunohistochemistry with a PSD-95 antibody recognizing aa 77-299 confirmed a severe decrease of PSD-95 (Figure 3D) in degenerating neurons of the ischemic tissue, identified in contiguous sections using the neurodegeneration specific marker Fluoro-Jade C (FJC, Figure 3E). We also examined in detail the cortical area separating the ischemic and non-ischemic tissues (Figure 3F) which is the predictable target of the designed neuroprotective strategy. Without intervention, this area might eventually degenerate to become ischemic tissue as the infarct expands from 5 to 24 h. Neurons in this area expressed different levels of PSD-95 and a correlation was found between condensed nuclei, characteristic of dying cells, and reduced PSD-95 levels (illustrated by neurons shown in panels a and b). In contrast, neurons expressing the highest levels of PSD-95 resembled those in the contralateral hemisphere (compare panel c with Figure 3D, panel a). Immunoblot analysis of the ischemic tissue (I) and the corresponding contralateral region (C) of the same animal or sham-operated animals confirmed and extended these observations (Figure 3G-J). Protein levels of the FL protein detected with PSD-95 Ct and Nt antobodies dramatically decreased in the infarcted region from very early time-points after brain damage (Figure 3G), being hardly detectable by 2.5 h (Figure 3H). This downregulation was concurrent with calpain activation in the ischemic area. Additionally, Ct and Nt-fragments of mobility and relative abundance similar to that previously found *in vitro* were strongly produced in the infarcted tissue as early as 2.5 h after cold-light irradiation. Ct-fragments accumulated later on, reaching maximum levels at 5 h (Figure 3I) or 24 h (Figure 3J) respectively for Ct-2 + Ct-3 or Ct-4. Compared to the *in vitro* results, fragments Ct-1, Nt-1 and Nt-2 presented a lower stability and were hardly detectable* in vivo*. Additional proteins identified in the ischemic tissue by the monoclonal antibody PSD-95 Ct (Figure 3G, upper panel asterisks) were similarly detected with an anti-mouse secondary antibody (Figure S4). This result suggests that they were due to early breakage of the BBB after ischemic damage as previously described 43, and leakage to the ischemic brain of IgG heavy (H) and light (L) chains, together with other serum proteins. In fact, in addition to the IgG chains, we detected in the ischemic tissue a protein of molecular weight similar to that of serum albumin which could be explained by a low cross-reactivity of this very abundant serum protein with the anti-mouse antibodies. In conclusion, these results demonstrate that PSD-95-processing is an early mechanism of regulation, not only induced by *in vitro* excitotoxicity but also in brain ischemia, where excitotoxicity takes place *in vivo*. They also suggest that prevention of PSD-95 calpain-processing might be explored as a novel strategy to approach stroke therapy.

### N-terminal sequencing identifies four calpain-processing sites inside PSD-95 intrinsically disordered regions (IDRs)

**Figure 4:**
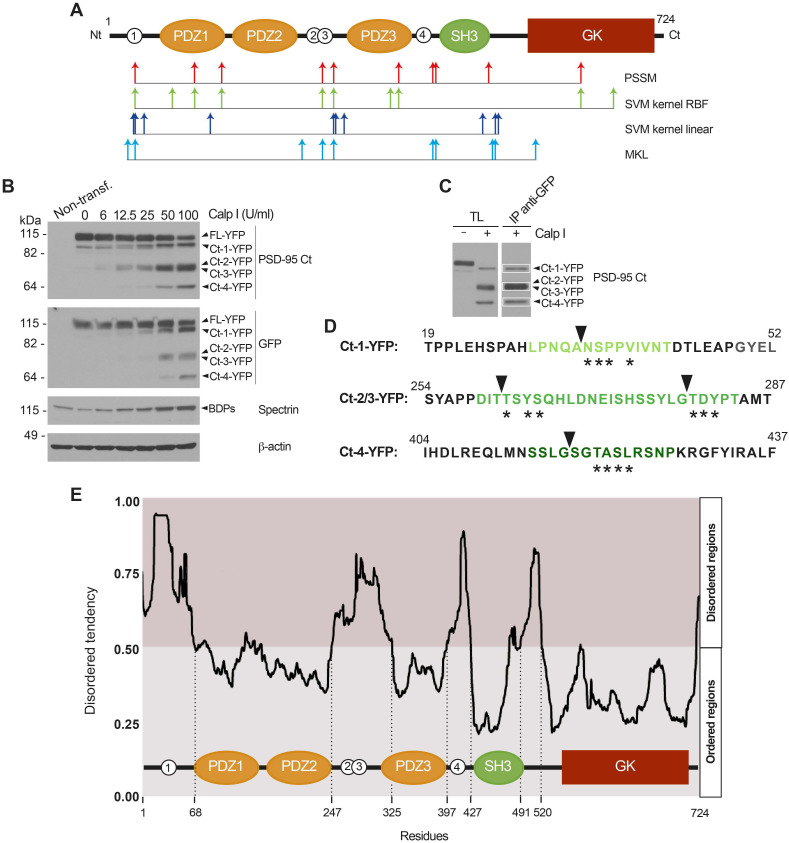
** N-terminal sequencing identifies four calpain-processing sites inside PSD-95 intrinsically disordered regions (IDRs).** (A) Putative calpain-cleavage sites predicted *in silico* by different algorithms. Below a diagram showing PSD-95 domains as before, arrows represent the 10 processing sites with the highest statistical scores for algorithms PSSM (red), SVM kernel RBF (green), SVM kernel lineal (dark blue) and MKL (light blue). (B) *In vitro* processing of fusion protein FL-YFP expressed in HEK293T cells. Extracts were digested with calpain I (6-100 U/ml) for 30 min and analyzed by immunoblot with antibodies PSD-95 Ct and GFP. YFP fused Ct-fragments were named in correspondence to the non-fused versions. Calpain activity was confirmed and ß-actin was a loading control. (C) Purification of YFP fused Ct-fragments for Edman sequencing, obtained by *in vitro* calpain-cleavage. Preparative amounts of previous total lysates (TL) were digested with calpain I (100 U/ml) before GFP immunoprecipitation. Analysis of a small fraction allowed selection of three bands for Edman sequencing. (D) Results of Edman sequencing. Asterisks denote coincident aminoacids and arrowheads indicate the cleavage sites. PSD-95 sequences later selected for inclusion in cell-penetrating peptides (CPPs) are highlighted in green. (E) Prediction of PSD-95 IDRs in relationship to protein domains. For simplicity, only one plot of the four metapredictions provided was represented (Metadisordermd2). Residues whose disorder probability is over 0.5 are considered as disordered and concentrate in inter-domain sequences.

To guide the design of CPPs able to specifically interfere with PSD-95 cleavage and preserve levels of this important protein in excitotoxicity and ischemia, it was important to establish the precise PSD-95 sequences cleaved by calpain. Such information would also confirm the PSD-95 domains that result uncoupled in excitotoxicity and suggest how this uncoupling might affect signal transduction. The mechanisms of substrate recognition and cleavage by calpain are largely unknown 44 and there is not a consensus linear recognition sequence. Instead, important determinants of substrate susceptibility are the secondary and tertiary protein conformations surrounding the cleavage sites 45 which often correspond to interdomains. In fact, *in silico* analysis of PSD-95 using several predictive algorithms for calpain-processing (CAMPDB 46, GPS-Calpain Cleavage Detector 47 and SitePrediction 48) indicated that the 10 predictions with the highest scores made by each algorithm mostly clustered in interdomain regions (Figure 4A) and, moreover, were compatible with results obtained by analysis of the Ct and Nt-fragments (Figure 1B-E; summarized in Figure 1F).

In an experiment previous to the isolation of the PSD-95 Ct-fragments required for Edman sequencing, we expressed a fusion protein of PSD-95 with the yellow fluorescent protein (YFP) bound to its C-terminus in heterologous cells. Extracts from the transfected cells were incubated with different amounts of calpain I and analyzed by immunoblot with PSD-95 Ct or GFP antibodies (Figure 4B). The molecular weight of the fragments obtained (105, 70 and 55 kDa) was compatible with fusion of YFP (27 kDa) to the Ct-fragments previously observed in neuronal extracts (Figure 2I-J). Then, in a preparative experiment, the total lysate (TL) was incubated with the maximum amount of calpain I tested before for complete PSD-95 cleavage, followed by immunoprecipitation (IP) of the fused Ct-fragments with GFP antibodies. The efficiency of immunoprecipitation was confirmed by immunoblot of a small aliquot with PSD-95 Ct antibodies (Figure 4C) before fractionation of the immunoprecipitated protein in preparative gels. Three bands corresponding to Ct-1-YFP, a pool of Ct-2-YFP plus Ct-3-YFP, and Ct-4-YFP (Figure 4C, white boxes) were collected and subjected to N-terminal sequencing by Edman degradation analysis.

For purified Ct-1-YFP, sequencing established N/A-S/V-P-M-Q/V as the N-terminus, a sequence not found in PSD-95. However, a unique sequence (NSPPV) containing four of these aminoacids could be found at PSD-95 N-terminus (aa34-38, asterisks in Figure 4D) immediately before the PDZ1 domain and after C3 and C5, PSD-95 cysteine residues which are modified by palmitoylation and contribute to synaptic clustering 49. Processing at this site would produce a truncated PSD-95 with molecular weight compatible with that of Ct-1 (Figure 1F). Analysis of the pooled fragments produced sequence T-D-Y-S, again absent in PSD-95. However, we found two nearby sequences with three coincidences: TSYS (aa262-265) and TDYP (aa280-283), located in the interdomain separating PDZ2 and PDZ3. Cleavage at these sites would generate two PSD-95 fragments of very similar molecular weight and compatible with those of Ct-2 and Ct-3 (Figure 1F). Finally, for Ct-4 the result obtained was Q-N-T/M-A/K-S-L and the closest PSD-95 sequence found was SGTASL (aa418-423), right downstream PDZ3 and part of the α-helix extension which is important for PDZ3 function and native fold stabilization 50. Interestingly, the four cleavage sites deduced by Edman sequencing were part of intrinsically disordered regions (IDRs) predicted inside the PSD-95 sequence using the online GeneSilico Metadisorder service 51 (Figure 4E). IDRs are central units of protein function and regulation due to their ability to establish multiple protein interactions 52 and can potentially act as weak signals for degradation 53. Globally, these results suggest that complete processing of PSD-95 by calpain in the four established sequences would produce three big fragments, one containing PDZ1 and PDZ2 and unable to bind the plasma membrane, another formed just by the PDZ3 domain and, finally, a third one containing domains SH3 and GK. However, results obtained *in vivo* also show incomplete PSD-95 processing at the PDZ3-SH3 linker, even at long times after brain damage, and production of the PDZ3-SH3-GK supradomain (Ct-2 and Ct-3, Figure 3G and I).

### Design and analysis of CPPs containing PSD-95 cleavage sequences as neuroprotectants for acute and chronic *in vitro* excitotoxicity

**Figure 5:**
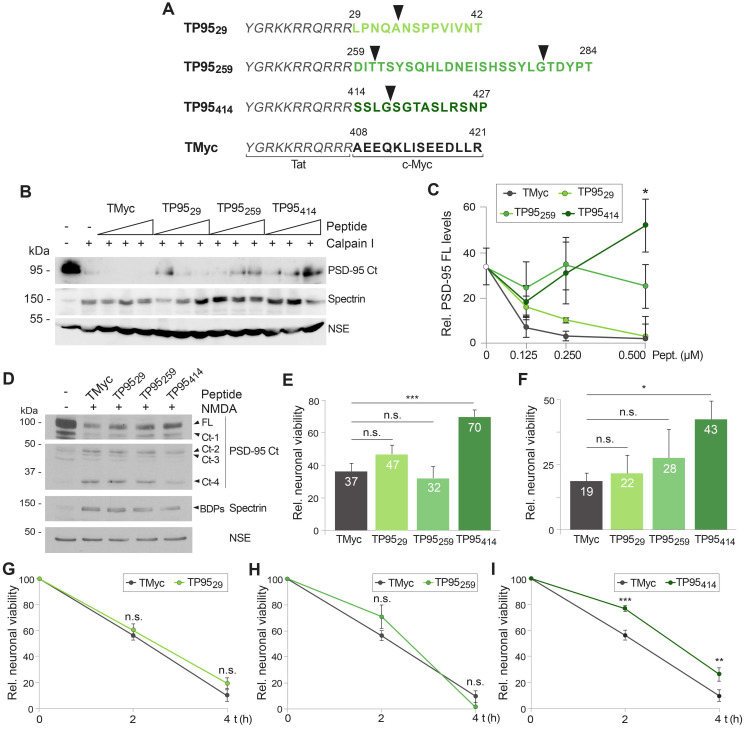
** Design and analysis of CPPs containing PSD-95 cleavage sequences as neuroprotectants for acute and chronic *in vitro* excitotoxicity.** (A) CPP design. Peptides contained Tat aa 47-57 (italic) followed by the indicated PSD-95 (green; TP95_29,_ TP95_259_ and TP95_414_) or c-Myc (black; TMyc) sequences. The four predicted calpain-cleavage sites are shown by arrowheads. (B) Dose-response analysis of the interference by the designed CPPs of PSD-95 processing induced by calpain *in vitro* digestion. Adult rat cortical extracts were incubated with calpain I (80 U/ml for 30 min) in the presence of 0.125, 0.250 and 0.500 µM of TMyc, TP95_29,_ TP95_259_ or TP95_414_ as indicated. (C) Quantitation of peptide effects on PSD-95 *in vitro* processing by calpain I. PSD-95 FL levels were expressed relative to those obtained in the undigested extracts. Means ± SEM (n = 3) were represented and analyzed by unpaired Student *t* test, comparing digestions in the presence of the PSD-95 peptides to those with TMyc. *p < 0.05. (D) Effect of TP95_29,_ TP95_259_ and TP95_414_ on downregulation of PSD-95 levels induced by acute NMDAR-overactivation. Cultures preincubated with PSD-95 peptides or TMyc (25 µM, 1 h) were treated with NMDA (100 µM, 1 h). After CPP and agonist removal, cultures proceeded for 23 additional hours with DL-AP5 (200 µM). Levels of PSD-95 FL and Ct-fragments at the end of this period were compared to untreated cells. (E) Effect of TP95_29,_ TP95_259_ and TP95_414_ pretreatment on neuronal viability induced by acute excitotoxicity. Cells were preincubated and treated as above and neuronal viability was established at the end of the 24 h period. Mean ± SEM values were represented relative to cultures without CPP or NMDA, arbitrarily given a 100% value (n =6). Analysis was performed by ANOVA test followed by LSD *post-hoc* test. n.s.: non-significant, ***p < 0.001. (F) Effect on neuronal viability of TP95_29,_ TP95_259_ and TP95_414_ added after induction of acute excitotoxicity. Cultures were treated with NMDA (100 µM, 15 min) without peptide preincubation. After agonist removal, cultures proceeded for 24 additional hours in the presence of TP95_29,_ TP95_259_ and TP95_414_ (25 µM) together with DL-AP5 (200 µM). Neuronal viability was established at the end of this period. Results were represented and analyzed as above (n = 6). n.s.: non-significant, *p < 0.05. (G, H and I) Effect of TP95_29,_ TP95_259_ and TP95_414_ on neuronal viability after chronic excitotoxicity. Cultures were preincubated with TMyc or PSD-95 CPPs (25 µM) and treated with NMDA (50 µM) for 2 or 4 h as before. Mean ± SEM values for each time-point were represented relative to those of neurons incubated with the same peptide but no NMDA (n = 9). Data were analyzed by a generalized linear model followed by a LSD *post-hoc* test. n.s.: non-significant, **p < 0.01, ***p < 0.001.

To preserve PSD-95 function in excitotoxicity and ischemia, we designed and characterized three CPPs based on the four established protein cleavage sites (arrowheads in Figure 5A). The generated CPPs contained the HIV-1 Tat basic domain, conferring membrane permeability and the capability of crossing the BBB to attached cargoes 23, fused to different stretches of the PSD-95 sequence containing the established cleavage sites (highlighted in different shades of green in Figure 4D). Specifically, TP95_29_ contained PSD-95 aa 29-42, TP95_259_ aa 259-284 and TP95_414_ aa 414-427 (Figure 5A). The detailed location of these sequences in relationship to the predicted PSD-95 IDRs is highlighted in Figure S5. As a negative control, we designed a similar Tat peptide with unrelated sequences corresponding to c-Myc (TMyc). We have previously demonstrated that TMyc is able to enter neurons but has no effects on neuronal viability both in basal or excitotoxic conditions 30 proving that Tat-based CPPs have no generic effects and can be used to test potential neuroprotective sequences.

First, we analyzed the capability of the designed peptides to prevent PSD-95 processing induced *in vitro* by purified calpain I in extracts prepared from adult brain cortex (Figure 5B-C), the intended target of neuroprotection in our stroke model. Cleavage of PSD-95 induced by calpain was partially prevented in a dose-dependent manner only by TP95_414_, while TP95_29_ and TP95_259_ had no significant effects compared to TMyc. Next, we characterized the peptides effects on cultured neurons by analyzing PSD-95 cleavage and neuronal death in the cellular model of acute excitotoxicity. Cultures were preincubated with TP95_29,_ TP95_259_ and TP95_414_ for 1 h before acute treatment with NMDA. This experimental paradigm tried to model an *in vivo* situation where a stroke patient received a peptide to protect still viable penumbra neurons ahead of a presumable secondary damage due to excitotoxicity which would provoke the expansion of the infarct core. After peptide and agonist removal, incubation proceeded in the presence of the NMDAR antagonist DL-AP5 (200 μM) to complete 24 h. At this time-point, levels of PSD-95 (Figure 5D) or neuronal viability (Figure 5E) were analyzed and compared to those of cultures treated with the control peptide. Results suggested that only peptide TP95_414_ was able to interfere PSD-95 calpain-processing and reduce the production of Ct-fragments in a cellular model of excitotoxicity (Figure 5D). Correspondingly, in the presence of TP95_414_, neuronal viability was only reduced by acute excitotoxicity to 70 ± 4% compared to control cultures without peptides or NMDA (Figure 5E). In contrast, viability was strongly decreased and reached similar values in cultures preincubated with TP95_29_, TP95_259_ or TMyc. Likewise, TP95_414_ was still able to prevent neuronal death when applied after induction of an irreversible acute excitotoxic damage (Figure 5F). Finally, the effect of the PSD-95 CPPs on neuronal viability was also analyzed after chronic NMDA treatment. Cultures were preincubated with peptides TMyc, TP95_29,_ TP95_259_ and TP95_414_ before treatment with NMDA for 2 or 4 h. Then, neuronal viability at these time-points was compared to cells incubated with the same peptide but no NMDA. No significant differences were found in cultures preincubated with TP95_29_ (Figure 5G) or TP95_259_ (Figure 5H) compared with cells treated with TMyc, which showed a marked time-dependent decrease in neuronal viability. In contrast, TP95_414_ increased neuronal viability at both evaluated times (Figure 5I) and, for example, reached values of 77 ± 2% at 2 h of treatment, significantly higher than those obtained for TMyc (56 ± 4%). Altogether, above results demonstrate that, differently from TP95_29_ and TP95_259_, peptide TP95_414_ is neuroprotective for both chronic and acute excitotoxic stimulus. They also suggest that prevention of neuronal death correlates with the interference of PSD-95 calpain-processing.

### TP95_414_ action preserves levels of PSD-95, neuronal viability and morphology in chronic excitotoxicity and PSD-95 cleavage is regulated by S/T phosphorylation

**Figure 6:**
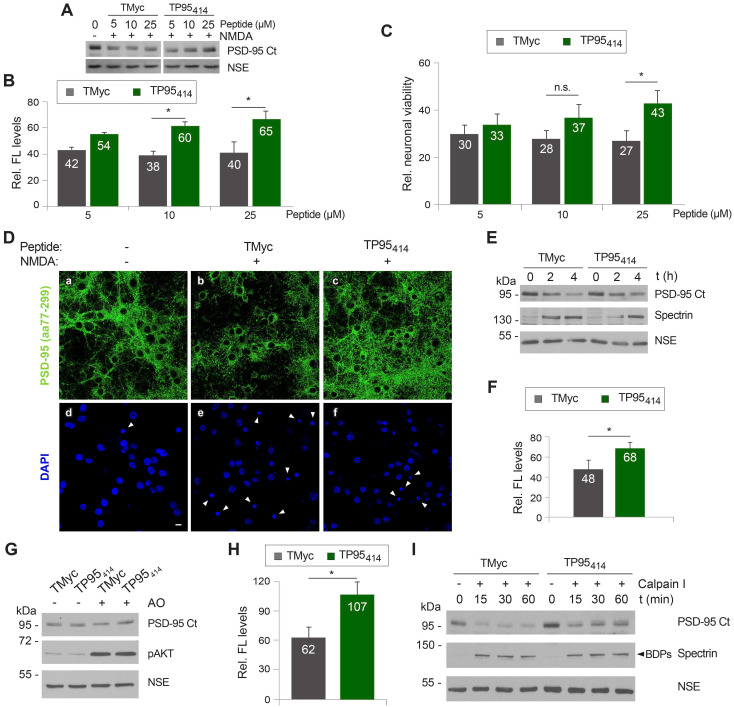
** TP95_414_ action preserves levels of PSD-95, neuronal viability and morphology in chronic excitotoxicity and PSD-95 cleavage is regulated by S/T phosphorylation.** (A-C) Dose-dependent TP95_414_ effects on PSD-95 downregulation and neuronal viability. Cultures preincubated for 1 h with different concentrations of TMyc or TP95_414_ (5, 10 and 25 µM) were treated with NMDA (50 µM, 4 h). A representative experiment showing the effects on PSD-95 is shown in panel A. Normalized PSD-95 FL levels (B) or neuronal viability (C) were expressed relative to values obtained in control cultures without peptide or NMDA and means ± SEM were represented (n = 3 or n = 6, respectively). Analysis was performed by ANOVA test followed by a LSD *post-hoc* test, and the comparison of TMyc or TP95_414_-treated cells for each peptide dose is shown. n.s.: non-significant, *p < 0.05. (D) Prevention by TP95_414_ of the characteristic morphological changes induced in neurons by excitotoxicity. Cultures were preincubated with TMyc or TP95_414_ (25 µM) and treated with NMDA for 2 h as before. Immunofluorescence was performed with antibodies recognizing aa 77-299 in PSD-95 (green) and nuclei stained with DAPI (blue). Arrowheads point to condensed nuclei indicative of neuronal death. Scale bar, 10 µm. (E) Time-course of TP95_414_ effects on PSD-95 downregulation. Cells preincubated with peptides (25 µM, 1 h) were treated for 2 or 4 h with NMDA as before. (F) Quantitation of TP95_414_ effects after 4 h of treatment. Normalized PSD-95 FL levels were represented relative to values obtained in cells incubated with the same peptide but no NMDA. Means ± SEM were given (n = 5). Results were analyzed by unpaired Student *t* test, comparing TMyc or TP95_414_-treated cells. *p < 0.05. (G) Effect of TP95_414_ on PSD-95 downregulation induced by S/T hyperphosphorylation. Cultures preincubated with TMyc or TP95_414_ (25 µM, 1 h) were treated with 1 µM okadaic acid (OA) for 30 min. Levels of PSD-95 FL in OA-incubated cells were compared to cultures with no inhibition of PP1 and PP2A. OA activity was confirmed with AKT S473 phospho-specific antibodies. (H) Quantitation of TP95_414_ effect on PSD-95 decrease induced by OA. Normalized PSD-95 FL levels were represented relative to values obtained in cells incubated with the same peptide but no OA. Means ± SEM were given (n = 5). Results were analyzed by unpaired Student *t* test, comparing TMyc or TP95_414_-treated cells. *p < 0.05. (I) Effect of TP95_414_ on the susceptibility of hyperphosphorylated PSD-95 to *in vitro* processing by calpain I. Extracts prepared from neurons preincubated with peptides and treated with OA as before were incubated with calpain I (50 U/ml) for 15, 30 or 60 min.

Once validated the feasibility of our neuroprotective approach, we decided to further characterize TP95_414_ effects to strengthen the correlation between PSD-95 preservation and neuronal survival. We analyzed in parallel PSD-95 levels (Figure 6A-B) and neuronal viability (Figure 6C) in primary cultures preincubated with different concentrations of TMyc or TP95_414_ before chronic NMDA treatment for 4 h. For the TMyc-incubated cultures, similar PSD-95 levels were reached in excitotoxicity at all peptide concentrations tested. In contrast, TP95_414_ significantly interfered with PSD-95 processing for peptide concentrations higher than 5 µM (Figure 6A-B). Analysis of neuronal survival showed that only a TP95_414_ concentration of 25 µM was able to significantly reduce neuronal death at this time-point in comparison to the same dose of TMyc (Figure 6C). Based on these results, we chose 25 µM as the optimum dose to analyzed TP95_414_ effects in excitotoxicity, as we could find statistical significant differences both in PSD-95 downregulation and neuronal death compared to the control peptide.

In a complementary experiment, we analyzed the effect of TP95_414_ on neuronal morphology and degeneration. Cortical cultures were preincubated with 25 µM TMyc or TP95_414_ before NMDA treatment and analyzed by immunofluorescence with PSD-95 antibodies and DAPI (Figure 6D). The characteristic morphological changes induced in neurons by excitotoxicity 35 were observed in cultures preincubated with TMyc, where a decrease of PSD-95 signal and its concentration in cell bodies was apparent, together with nuclei structural changes such as chromatin condensation, nuclei shrinkage and formation of apoptotic bodies (Figure 6D, arrowheads). In contrast, PSD-95 levels in cultures preincubated with TP95_414_ were comparable to those of the untreated cells, the protein being distributed both in cell bodies and along neuronal processes. Signs of neuronal degeneration were also partially reverted by TP95_414_. Finally, we did a time-course experiment to compare the decrease of PSD-95 in cultures pretreated with 25 µM of TMyc or TP95_414_ before chronic NMDAR-overactivation (Figure 6E-F). After 4 h of NMDA treatment, cells incubated with TP95_414_ had significantly higher PSD-95 levels than cultures treated with TMyc (respectively 68 ± 6% and 48 ± 9%). The differences were not significant for 2 h of NMDA treatment, although there was already an effect on neuronal viability at this time-point (Figure 5I). In conclusion, we find a good correlation between the interference of PSD-95 processing by TP95_414_ and prevention of the morphological changes that precede neuronal death in excitotoxicity. Moreover, the neuroprotective effects of TP95_414_ are dose and time-dependent.

Substrate phosphorylation often regulates calpain-processing 54, 55 and, interestingly, several S/T residues are located in or nearby the PSD-95 processing site at the PDZ3-SH3 linker (Figure 4D). Thus, we decided to approach if excitotoxicity-induced PSD-95 degradation might be regulated by phosphorylation and how TP95_414_ could affect such regulation. Cultures were pretreated with TMyc or TP95_414_ as above before inhibition of protein S/T phosphatases 1 (PP1) and 2A (PP2A) by incubation with okadaic acid (OA) for 30 min (Figure 6G). Increased phosphorylation of S/T residues was confirmed by analyzing S473 phosphorylation of protein AKT. Quantification of the results proved that augmented protein phosphorylation very rapidly reduced PSD-95 stability, probably due to enhanced action of endogenous calpain on hyperphosphorylated PSD-95. Levels of this protein decreased to 62 ± 10% in cultures treated with TMyc and OA compared to those only incubated with this peptide (Figure 6H). Interestingly, preincubation with TP95_414_ prevented this OA-induced decrease in PSD-95 levels. In addition, protein extracts prepared from cells treated as before with TMyc and OA were more susceptible to *in vitro* degradation by calpain I (50 U/ml) compared to lysates from cells treated with TP95_414_ and OA (Figure 6I). Altogether, these results suggest that PSD-95 calpain-cleavage is regulated by S/T phosphorylation and that TP95_414_ interferes this processing.

### TP95_414_ reduces neurological damage induced by permanent ischemia

**Figure 7:**
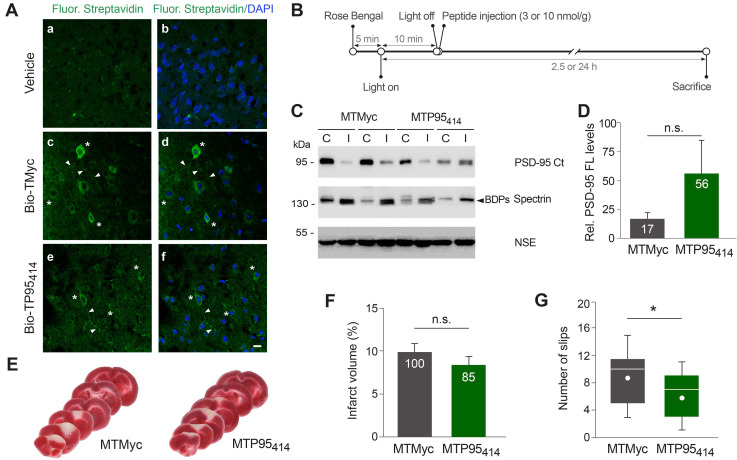
** TP95_414_ reduces neurological damage induced by permanent ischemia.** (A) Confirmation of TMyc and TP95_414_ delivery to mice cortex. Biotinylated TMyc (Bio-TMyc) and TP95_414_ (Bio- TP95_414_, 3 nmol/g) were retro-orbitally injected and detected in coronal sections of animals sacrificed 30 min later, stained with Fluorescein Streptavidin and DAPI. Representative confocal microscopy images of cortical areas corresponding to single sections show peptide distribution in cell bodies (asteriks) and neurites (arrowheads) of cortical neurons, absent in vehicle-injected animals. Scale bar, 10 µm. (B) Timeline to analyze the *in vivo* effects of MTMyc and MTP95_414_. Mice subjected to ischemia as before were retro-orbitally injected with CPPs (10 or 3 nmol/g) 10 min after damage initiation. Animals were sacrificed at 2.5 h of photothrombosis for immunoblot analysis or 24 h for other experiments. (C) Representative analysis of PSD-95 at 2.5 h of damage in animals injected with 3 nmol/g of MTMyc or MTP95_414_. Comparison of the infarcted area (I) and the corresponding contralateral region (C) for two different animals in each experimental group is shown. (D) Quantitation of PSD-95 levels_._ Means ± SEM of normalized PSD-95 levels in the infarcted area relative to the corresponding contralateral region were represented (n = 6) and results analyzed by Student's *t*-test. n.s. = non-significant. (E) Representative 1 mm brain coronal slices stained with TTC after 24 h of insult corresponding to animals injected with MTMyc or MTP95_414_ (3 nmol/g). (F) Infarct volume of animals injected with MTMyc or MTP95_414_ (3 nmol/g) at 24 h of damage. Volumes were expressed as a percentage of those of the hemisphere and means ± SEM are given (n = 15). Differences were analyzed by Student's *t*-test. n.s. = non-significant. Results for MTP95_414_ are also expressed as percentage of the infarct volume in animals injected with MTMyc, arbitrarily considered a 100% value. (G) Evaluation of balance and motor coordination of animals injected with 3 nmol/g of MTMyc or MTP95_414_. Number of contralateral hind paw slips were measured after 24 h of damage. Data spread is presented by box and whisker plots showing interquartile range, median, minimum and maximum values (n = 15). Means (dots) were also calculated and analyzed by Student's *t*-test. *p < 0.05.

We decided next to investigate if TP95_414_ could also act as a neuroprotectant in ischemia. First, we analyzed if biotinylated TP95_414_ (Bio-TP95_414_) was able to reach the brain area that will be damaged in our stroke model. We established that Bio-TP95_414_, as well as biotinylated TMyc (Bio-TMyc), were efficiently distributed in cell bodies and neurites of cortical neurons (Figure 7A). Thus, these peptides are able to cross the BBB in undamaged brain, reach the cortex and enter brain cells. Disruption of the BBB is a pathophysiological hallmark of ischemic stroke 43, thus brain distribution of these CPPs could be further increased after damage. Next, and before starting the *in vivo* analysis of TP95_414_ neuroprotective potential, we improved the plasma stability of TMyc and TP95_414_ by modification of their terminal sequences to mimic the natural protein structure. Thus, we generated peptides MTMyc and MTP95_414_ respectively by acetylation and amidation of their N-ter and C-ter sequences (Table S1). Using primary cultures, we validated the effects of MTP95_414_ on neuronal survival in basal and excitotoxic conditions and found no significant differences with unmodified TP95_414_ (Figure S6). After that, we injected MTP95_414_ in mice 10 min after the initiation of brain damage by photothrombosis as summarized in Figure 7B. To evaluate the relevance of this experimental approach for clinical therapy, it is important to consider the different biological timescales of rodents and humans 56. These differences are particularly significant when studying acute disorders of the CNS, such as stroke, where changes occur rapidly over time and the therapeutic window can be easily missed. The early intervention in mouse after damage might model the treatment of patients several hours after the primary unpredictable brain damage. Initially, we chose a peptide concentration of 10 nmol/g because, in experiments designed to analyze dose-translation between species, this was the nerinetide concentration required in mice ischemia to obtain an effective neuroprotection comparable to that achieved in rat models with 3 nmol/g 57. However, in preliminary experiments, we found that MTP95_414_ was not neuroprotective in these conditions (data not shown). We reasoned that PSD-95 stabilization in ischemia might have dual effects on neuronal survival and that excessive levels of PSD-95 might be deleterious. Thus, we repeated these experiments lowering MTP95_414_ dose to 3 nmol/g. In these conditions, MTP95_414_ showed a tendency to reduce the downregulation of PSD-95 induced in the ischemic region early after damage (2.5 h) although the differences found with MTMyc-treated animals did not reach statistically significance (Figure 7C-D). Next, we examined the long-term effects (24 h) that MTP95_414_-treatment might have on infarct volume. We performed TTC staining of coronal sections (Figure 7E) and established infarct volume (Figure 7F). In MTP95_414_-treated animals, infarct volume was 8 ± 1% relative to total hemisphere volume, a value smaller than that obtained in the MTMyc group (10 ± 1%). Although this represents a 15% reduction in infarct size due to MTP95_414_-treatment, this difference was not statistically significant (Figure 7F). Remarkably, this moderate decrease of infarct volume correlated with a significant improvement in balance and motor coordination in the beam walking test. We found a decrease of 33% in the number of slips due to MTP95_414_-treatment compared to MTMyc animals (mean values respectively 6 ± 1 and 9 ± 1, Figure 7G). Altogether, these results demonstrate that the neuroprotective peptide MTP95_414_ strongly reduces neurological damage when used at a dose of 3 nmol/g in a severe model of permanent brain ischemia. Additionally, our data unveil the importance of a correct balance of this postsynaptic protein for neuronal survival after brain damage and assist in the rational development of therapies for stroke treatment.

## Discussion

Herein, we report the design and characterization of a neuroprotective CPP, TP95_414_, which prevents downregulation of scaffold protein PSD-95 in correlation with increased viability of neurons subjected to acute or chronic NMDAR-overactivation. This peptide was designed after a rational characterization of the mechanisms of PSD-95 downregulation induced by excitotoxicity. Levels of this major component of excitatory synapses 17 are strongly and specifically decreased by excitotoxic damage, which does not alter other DLG proteins such as SAP-97 or SAP-102. In contrast, PSD-95 downregulation is concurrent with that of additional components of NMDAR-complexes such as neurotrophin receptor TrkB-FL (as shown in this work) or GluN2A/B NMDAR-subunits 3, likewise calpain-substrates 3, 6. This concerted decline profoundly alters the complex network of protein interactions normally established at the PSD and affects critical survival pathways, resulting in neurotoxicity.

We have recently demonstrated that prevention of the downregulation of a specific calpain-substrate such as TrkB-FL results in neuroprotection, both in excitotoxicity *in vitro* or infarcted brain 30. Therefore, it was reasonable to propose that interference of PSD-95-processing induced by NMDAR-overactivation might also constitute a novel therapeutic approach for stroke treatment. To design such PSD-95-targeted neuroprotective strategy, we analyzed the identity and stability of the calpain-processing fragments generated *in vitro* or* in vivo*. The combination of immunoblot analysis, *in silico* studies, and fragment purification and sequencing, identified four cleavage sites between PSD-95 positions 33-34, 259-260, 279-280 and 417-418. In accordance to a general trend of calpain activity, these processing sites are located at specific linker regions separating PSD-95 domains: upstream the PDZ1 and downstream PDZ2 or PDZ3, inside sequences corresponding to major predicted IDRs 51. These are flexible polypeptide sequences unable to fold into structured domains although critical to different cell functions 58, including signal transduction and formation of protein complexes or structural protein scaffolds. IDRs often present potential phosphorylation sites that, once modified, might modulate their conformation, protein-protein interactions and transition between ordered and disordered states 59. Notably, IDRs can also perform as weak signals for intracellular protein degradation 53 as found here for PSD-95.

We propose that the effects of excitotoxic PSD-95 processing on neuronal survival and functioning are due to a strong decrease of the full-length protein, fracture of PSD-95 supertertiary structure and production of a set of stable fragments of unknown function. PSD-95 dysfunction has been associated before with neuropsychiatric disorders such as schizophrenia, autism and intellectual disorders 60, 61. Deficiency of PSD-95 in development provokes a concomitant overactivation of NMDARs and D1 receptors that makes neurons more susceptible to excitotoxicity and causes neurological impairment 62. PSD-95 knockout also changes NMDAR and AMPAR expression and function in the medial prefrontal cortex provoking behavior alterations 63. Finally, acute PSD-95 knockdown in mature neurons similarly alters synaptic transmission and neuronal vulnerability 28. Altogether, these data suggest that the PSD-95 deficiency induced by excitotoxicity might have by itself an important impact on neuronal function. Additionally, PSD-95 processing between residues 33-34 produces a truncated protein (Ct-1 fragment) lacking palmitoylated residues C3/C5, essential for protein stabilization at the PSD and synaptic clustering 49. Further cleavage between aminoacids 259-260 and/or 279-280 uncouples the PDZ1-2 supramodule (Nt-3 and Nt-4 fragments) and P-S-G (Ct-2 and Ct-3 fragments), the latter lacking the specific protein interactions normally established by PDZ1-2 and required, in addition to C3/C5, for PSD-95 clustering and stability 64. Finally, additional effects will be probably due to partial processing between residues 417-418, inside the PDZ3-SH3 linker, breaking the structural P-S-G supradomain and producing the SH3-GK tandem (Ct-4 fragment).

Once established the PSD-95 processed sequences, we designed three CPPs with potential to specifically interfere this cleavage. Compared to generic calpain inhibitors, which could block physiological protease actions and produce unwanted side-effects 65, we chose to inhibit processing of a particular substrate that, additionally, only occurs under pathological conditions and would not interfere with physiological PSD-95 functions. Peptides TP95_29_ and TP95_259_ were not able to maintain neuronal levels of PSD-95, one possibility being that the selected sequences were insufficient to provide the three-dimensional conformation required for calpain recognition. In contrast, TP95_414_, containing PSD-95 sequences less efficiently processed by calpain, could partially preserve the P-S-G supradomain and also the complete protein. This peptide contains residues located inside the PDZ3-SH3 linker (404-428), a very flexible IDR described as critical for P-S-G supertertiary structure 12. The central linker segment mediates a weak and dynamic PDZ3-SH3 association that can be reverted by PDZ3-binding to PDZ-ligands (e.g. CRIPT) or electrostatic repulsion upon linker phosphorylation in residues S415 and S418 66, resulting in increased PDZ3 mobility relative to the SH3-GK tandem. Our preliminary results suggest that PSD-95 processing might be regulated by phosphorylation, as found for other calpain-substrates 54, 55, and that PSD-95 sequences present in TP95_414_ participate in this regulation. Thus, phosphorylation of linker residues might loosen domain packaging of the P-S-G supramodule and make this site more accessible for calpain, this protease cleaving right before S418. Processing might then irreversibly interfere with PDZ3 and SH3-GK coupling and the formation of PSD-95 homo- and hetero-oligomers 16. Peptide TP95_414_ could prevent this processing either directly, acting as a mock calpain-substrate and blocking calpain activity over this particular site, or indirectly, by altering phosphorylation of PSD-95 residues S415 and/or S418. Anyway, recovery of substantial amounts of complete protein by TP95_414_ action suggests that calpain activity on PSD-95 might be hierarchically organized and that regulated cleavage at PDZ3-SH3 linker determines further protein processing. Thus, treatment with TP95_414_ would primarily prevent cleavage between residues 417-418 and, secondarily, that in positions 33-34, 259-260 and 279-280.

We propose that PSD-95 supramodules have opposite effects on neuronal survival and while PDZ1-2 mediates neurotoxic functions, P-S-G is neuroprotective. Thus, dissociation by nerinetide of GluN2B-PSD-95-nNOS ternary complexes formed by PDZ1-2 is neuroprotective in excitotoxicity and ischemia 20-22, 24, conditions that uncouple the PDZ1-2 supramodule from the rest of the protein due to PSD-95 processing. Meanwhile, the alternative and complementary approach developed here results in neuroprotection by primarily maintaining the levels of the P-S-G supramodule and, secondarily, those of the complete protein. Several observations indicate that this peptide might be highly relevant to human stroke therapy. First, we have demonstrated that TP95_414_ is able to cross the BBB of undamaged brain, reach the cortex and enter brain cells where it is detected in the cell bodies and neurites of cortical neurons. Peptide delivery might be further increased in the ischemic brain, due to early BBB disruption after damage 43 and stroke promotion of excitotoxicity-induced endocytosis 67, an important cell entry route for Tat-derived peptides 23. The experimental mouse model developed here was aimed to make peptide TP95_414_ available to cells of the ischemic penumbra 10 min after the primary insult (photothrombotic ischemia) but before the secondary excitotoxic damage could affect them. Considering the different biological timescales of rodents and humans 56, these conditions might simulate a clinical situation where peptides are provided to patients several hours after the primary insult to neuroprotect the functionally impaired tissue at risk of infarction by interference of PSD-95 processing and neuronal death. In fact, we show in this work that TP95_414_ tends to prevent PSD-95 downregulation and reduce infarct size in a severe model of permanent ischemia, where a relatively narrow ischemic penumbra 68 challenges the efficacy of neuroprotective strategies. In spite of the modest decrease of infarct size, TP95_414_ is still able to significantly improve the neurological function in treated animals, suggesting that this peptide might exert additional actions on other brain cell populations supporting synaptic transmission. It will be also interesting to investigate the neuroprotective efficacy of TP95_414_ in alternative models of transient ischemia having a wider ischemic penumbra. Finally, recovery by TP95_414_ action of synaptic signaling and function might be also important for treatment of other important neurological conditions different from stroke, likewise associated to excitotoxicity.

## Supplementary Material

Supplementary figures and tables.

## Abbreviations

AMPAR: α-amino-3-hydroxy-5-methyl-4-isoxazolepropionic acid receptor
Ara C: cytosine ß-D-arabinofuranoside
BBB: blood-brain barrier
BDNF: brain-derived neurotrophic factor
BDPs: breakdown products
CiIII: calpain inhibitor III
CPP: cell-penetrating peptide
CRIPT: cysteine-rich PDZ-binding protein
DA: dopamine
DLG: disc large homologue
GK: guanylate kinase-like
IDR: intrinsically disordered region
MCAO: middle cerebral artery occlusion
NMDAR: N-methyl-D-aspartate type of glutamate receptor
nNOS: nitric oxide synthase
NSE: neuron-specific enolase
OA: okadaic acid
PDZ: postsynaptic density protein-95/discs large/zonula occludens-1
PP: protein S/T phosphatase
PSD-95: postsynaptic density protein-95
SAP: synapse-associated protein
SH3: Src homology 3
TBS: Tris-buffered saline
TrkB: neurotrophin receptor tropomyosin-related kinase B
TrkB-FL: full-length receptor
