# Supplementary material for: A novel cell-penetrating peptide targeting calpain-cleavage of PSD-95 induced by excitotoxicity improves neurological outcome after stroke
Source: Theranostics. 2021 May 3;11(14):6746–65. doi: 10.7150/thno.60701 (PMC8171078; doi:10.7150/thno.60701)
Supplement: Supplementary file 1 — Supplementary figures and tables. [file thnov11p6746s1.pdf]

Supplementary information

Supplementary methods

All reagents, cell-penetrating peptides (CPPs), biological models, software and algorithms used in the study are detailed in Table S1 (see below).

Mouse model of ischemia by photothrombosis

Permanent focal ischemia was induced by microvascular photothrombosis in the cerebral cortex of adult male Balb/cOlaHsd mice (25-30 g; 8-12 weeks of age; Harlan Laboratories, Boxmeer, The Netherlands). Mice were anesthesized with isoflurane (5% for induction, 2% for maintenance in oxygen, Abbot Laboratories, Madrid, Spain), and then placed in a stereotaxic frame (Narishige Group, Tokyo, Japan). Body temperature was maintained at 36–37°C using a self-regulating heating blanket (Cibertec, Madrid, Spain). A midline scalp incision was made, the skull was exposed and both Bregma and Lambda points were identified. A cold-light (Schott KL 2500 LCD, Schott Glass, Mainz, Germany) with a fiber optic bundle of 1.5 mm in diameter was centered using a micromanipulator on the right side, at 0.2 mm anterior and 2.0 mm lateral (+0.2 AP, +2 ML) relative to Bregma. Afterwards the photosensitive dye Rose Bengal (7.5 mg/ml, prepared in sterile saline; Sigma-Aldrich) was administered by retro-orbital injection of the venous sinus, for intravenous (i.v.) vascular access, to a body dose of 20 mg/kg. Five min later, the brain was illuminated (600 lms, 3000K) through the intact skull for 10 min. The areas underneath this stereotaxic position that resulted irradiated were the primary motor cortex and the primary somatosensory cortex (hindlimb and forelimb), according to the Paxinos mouse brain atlas. In sham-operated animals, used as additional control in immunoblot experiments, the procedure was the same except for the omission of the irradiation step. After completion of the surgical procedure, the incision was sutured and mice were

allowed to recover. For immunoblot analysis, animals were sacrificed 2.5, 5 or 24 h after damage induction by CO_2 inhalation followed by cervical dislocation and their brains were sectioned into serial 2-mm-thick coronal slices using a mouse brain matrix (Stoelting, Wood Dale, IL, USA). Slices were briefly stained (4 min) with a cold 2% solution of triphenyltetrazolium chloride (TTC, Sigma-Aldrich) to avoid endogenous postmortem calpain activation. The unstained area of the cerebral cortex in the ipsilateral hemisphere (defined as infarcted tissue, I), as well as the corresponding region in the contralateral hemisphere (C), was dissected to prepare protein lysates. For assessment of infarct volume, animals were sacrificed as before 24 h after damage induction and their brains were sectioned into serial 1-mm-thick coronal slices using a mouse brain matrix as before. Slices were then completely stained with 2% TTC at room temperature for 10 min, and fixed in 4% paraformaldehyde before scanning of both rostral and caudal sides.

Primary culture of rat cortical neurons

Dissected cerebral cortices from 18-day-old Wistar rat embryos (E18) were mechanically dissociated in culture medium (Minimum Essential Medium) supplemented with 22.2 mM glucose, 0.1 mM glutamax, 5% fetal bovine serum, 5% donor horse serum, 100 U/ml penicillin and 100 μg/ml streptomycin. The cell suspension was seeded at a density of $1x10^6$ cells/ml in the same medium using plates previously treated with poly-L-lysine (100 μg/ml) and laminin (4 μg/ml) overnight at 37 °C.

Culture and transfection of HEK293T cells

Human embryonic kidney 293T cells (HEK293T) were cultured at 37 °C in Dulbecco's modified Eagle's medium (Invitrogen-Live Technologies), supplemented with 10% fetal bovine serum, 2 mM glutamine and 100 U/ml of penicillin/streptomycin in an humidified atmosphere containing 5% CO_2. Transfection was performed in serum-free medium with

Lipofectamine 2000 reagent and, 4 h after addition, liposomes were removed and cells fed with supplemented medium and maintained in culture for 48 h.

Western blot (WB) analysis

Cultures and brain tissue were lysed in RIPA buffer (50 mM Tris-HCl pH 8, 150 mM NaCl, 1% sodium deoxycholate, 1% NP-40, 1 mM DTT and 0.1% SDS for cultures or 1% SDS for tissue) containing protease and phosphatase inhibitors (Complete protease and PhosSTOP phosphatases inhibitor cocktail tablets, Roche). Protein concentration was established with BCA Protein Assay Kit (Thermo Fisher). Total cell-lysates were denatured in SDS-sample buffer followed by heating at 95° for 5 min. Equal amounts of total cell-lysates were resolved in Tris-Glycine SDS-PAGE and transferred on to a nitrocellulose membrane (GE Healthcare). Membranes were stained with a Ponceau S solution to check for protein transference efficacy. After blocking with a 5% nonfat dry milk solution in Tris-buffered saline (TBS) with 0.05% Tween-20, membranes were incubated overnight at 4°C with primary antibodies, and then washed and incubated with appropriate anti-rabbit or anti-mouse peroxidase-conjugated secondary antibodies (Santa Cruz Biotechnology) for 1 h at room temperature. Finally, immunoreactivity was visualized using Clarity Western ECL Blotting Substrate (BioRad) and band intensity was quantified by densitometric analysis (ImageJ or Adobe Photoshop). The levels of the protein of interest were normalized using those of neuron-specific enolase (NSE) present in the same sample and expressed relative to values obtained in their respective controls, arbitrarily given a 100% value. NSE was used as a neuronal loading control since it is not affected by NMDA treatment. In contrast, the activation of calpain induced by excitotoxicity was confirmed by analyzing the formation of characteristic breakdown products (BDPs; 150 and 145 kDa) from spectrin, a standard substrate of this protease.

Multiple independent experiments were carried out and quantitated as detailed in the figure legends.

Immunocytochemistry (ICC)

Primary cultures grown as indicated before on poly-D-lysine/laminin (Sigma-Aldrich) coated coverslips were fixed after treatment with 4% paraformaldehyde in PBS for 30 min and then washed with PBS. Cells were blocked and permeabilized for 30 min at room temperature with 1% BSA, 0.1% Triton X-100 in PBS. Afterwards coverslips were incubated overnight at 4°C with antibodies diluted in the same solution recognizing the PSD-95 region compromised between aminoacids 236-308 or 77-299, panTrkB and synapsin I, used at the dilutions indicated in Table S1. Detection was achieved with secondary antibodies conjugated to Alexa Fluor 488 or Alexa Fluor 546 before mounting with Prolong Gold. All confocal images are single sections acquired using an inverted Zeiss LSM 710 laser confocal microscope (Jena, Germany) with a 40 x or 63×Plan-Apochromatic oil immersion objective and were normalized for each color separately. Images were processed for presentation with ImageJ (NIH Image).

Immunohistochemistry (IHC) and Fluoro-Jade C staining

Mice were deeply anesthetized 5 h after brain damage and intracardially perfused with cold PBS and 4% paraformaldehyde in PBS. Brains were post-fixed in the same fixative at 4°C for 24 h and cryoprotected in 30% sucrose for 48 h at 4°C. Coronal frozen sections (30 µm thick) were obtained using a cryostat (Leica, Heidelberg, Germany) and the infarcted tissue was identified in some of them with cresyl violet (Nissl staining). Sections adjacent to those stained with Nissl were incubated with blocking solution (10% goat serum, 0.5% Triton X-100 in PB) in flotation for 1 h at room temperature and then with an anti-mouse IgG H&L antibody (Fab fragment) diluted in 5% goat serum, 0.5% triton X-100 in PB for 18 h at 4°C. This step tried to decrease background due to anti-mouse secondary antibodies

reaction with mouse antibodies leaked to the ischemic area as a consequence of early BBB breakage induced by damage. After washing, slides were incubated overnight at 4ºC with the antibody that recognizes PSD-95 aa 77-299, followed by Alexa Fluor 546- or Alexa Fluor 488-conjugated goat anti-mouse secondary antibodies to visualize the primary antibodies and DAPI (5 μg/ml) to stain DNA. The dilutions employed for these antibodies are indicated in Table S1. Specific labeling of degenerating neurons and cell nuclei was performed in adjacent sections by Fluoro-Jade C staining. To that, sections were immersed in a basic alcohol solution (1% NaOH in 80% ethanol) for 5 min, rinsed for 2 min in 70% ethanol and for 2 additional min in distilled water. Next, they were incubated in a 0.06% potassium permanganate solution for 10 min, rinsed for 2 min in distilled water, and transferred for 10 min to a fresh solution of 1 μg/ml Fluoro-Jade C in 0.1% acetic acid containing 0.5 μg/ml DAPI. Sections were then washed in distilled water, mounted on slides, dried for 15 min at 37ºC on a hot plate, air dried overnight and then cover slipped with DPX. Confocal images were acquired with a 63 x Plan-Apochromatic oil immersion objective and processed for presentation as above described.

RNA extraction and quantitative RT-PCR

Total RNA from primary cultures was extracted using the RNeasy mini kit (Quiagen, Germantown, MD, USA). The reverse transcription reaction was performed using degenerate primers and the "High Capacity cDNA Reverse Transcription Kit". The PCR reactions contained 5 ng of cDNA and 300 nM of the specific oligonucleotides indicated in Table S1. A 7900HT FAST Real Time PCR System (Applied Biosystems, Foster City, CA; USA) was used. The samples were denatured at 95 ° C for 10 min, followed by 40 cycles of 15 s at 95 °C and 60 s at 60 °C. The level of PSD-95 mRNA was normalized to that of 18S rRNA present in the same sample, and represented as relative values to those obtained in the untreated cells, arbitrarily considered as 100%.

Table S1. Description of reagents or resources used in the study

**REAGENT or RESOURCE**	**SOURCE**	**IDENTIFIER**
**Antibodies**		
Mouse monoclonal anti-**β-actin** (1:15000)	Sigma-Aldrich	Cat#A5441 RRID:AB_476692
Rabbit polyclonal anti-pSer473-**AKT** (1:1000)	Cell Signalling Technology	Cat#9271 RRID:AB_329825
Goat polyclonal anti-**GluN2A** NMDAR-subunit (1:250)	Santa Cruz Biotechnology	Cat# sc-1468; RRID:AB_670223
Mouse monoclonal anti-**GluN2B** NMDAR-subunit (1:500)	Antibodies Inc.	Cat#75-097; RRID:AB_10673405
Rabbit polyclonal anti-**GFP** (1:500)	Invitrogen	Cat#A6455; RRID:AB_221570
Rabbit polyclonal anti-neuronal-specific enolase (**NSE**) (1:20000)	Millipore	Cat#AB951; RRID:AB_92390
Rabbit polyclonal anti-**P53** (1:500)	Santa Cruz Biotechnology	Cat#sc-6243; RRID:AB_653753
Rabbit polyclonal anti-**PSD-95 (aa50-150)** (1:1000)	Abcam	Cat#ab18258; RRID:AB_444362
Mouse monoclonal anti-**PSD-95 (aa77-299)** (ICC: 1:1000; IHC: 1:500)	Antibodies Inc.	Cat#75-028; RRID:AB_2292909
Mouse monoclonal anti-**PSD-95 (aa236-308)** (WB 1:1500; ICC: 1:1000)	Chemicon	Cat#MAB1596; RRID:AB_2092365
Mouse monoclonal anti-**PSD-95 (aa353-504)** (1:250)	BD Bioscience	Cat#610495; RRID:AB_397861
anti-**SAP-97** (1:1000)	UC Davis/NIH NeuroMab Facil.	Cat# 75-030; RRID:AB_2091920
anti-**SAP-102** (1:250)	UC Davis/NIH NeuroMab Facil.	Cat# 75-058; RRID:AB_2261666
Mouse monoclonal anti-**spectrin** alpha chain (1:20000)	Millipore	Cat#MAB1622; RRID:AB_94295
Rabbit polyclonal anti-**synapsin I** (1:1000)	Chemicon	Cat# AB1543; RRID:AB_2200400
Rabbit polyclonal anti-**panTrkB** (WB: 1:500; ICC: 1:100)	Santa Cruz Biotechnology	Cat# sc-8316; RRID:AB_2155274
Goat anti-mouse-HRP (1:5000)	Santa Cruz Biotechnology	Cat# sc-2005; RRID:AB_631736
Goat anti-rabbit-HRP (1:5000)	Santa Cruz Biotechnology	Cat# sc-2004; RRID:AB_631746
Donkey anti-goat-HRP (1:5000)	Santa Cruz Biotechnology	Cat# sc-2056; RRID:AB_ 631730
Goat F(ab) anti-mouse IgG H&L (1:1000)	Abcam	Cat#ab6668; RRID:AB_955960
Goat anti-mouse IgG Alexa Fluor 488 (1:500)	Molecular Probes	Cat# A-11029; RRID:AB_138404

Goat anti-rabbit IgG Alexa Fluor 546 (1:500)	Molecular Probes	Cat# A-11035; RRID:AB_143051
Goat-anti-mouse IgG Alexa Fluor 546 (1:500)	Molecular Probes	Cat#A-11030; RRID:AB_144695
**Chemicals, Peptides, and Recombinant Proteins**		
**AraC** (used at 10 µM)	Sigma-Aldrich	Cat#C1768; CAS: 147-94-4
**Calpain I** (5-80 U/ml)	Sigma-Aldrich	Cat# 208712
**Calpain II** (50-200 U/ml)	Sigma-Aldrich	Cat#208715
Calpain inhibitor III (**CiIII**) (used at 10 µM)	Calbiochem	Cat#208722; CAS: 88191-84-8
**Calpeptin** (used at 10 µM)	Calbiochem	Cat#03-34-0051; CAS: 117591-20-5
**DAPI** (used at 0.5 and 5 µg/ml)	Molecular Probes	Cat#D1306
**DL-AP5** (used at 200 µM)	Tocris	Cat#0105
**DPX** Mountant for histology	Sigma-Aldrich	Cat#06522
**Fluorescein streptavidin** (used at 1 µg/ml)	Vector Labs	Cat#SA-5001-1
**Fluoro-Jade C** (FJC, used at 1 µg/ml)	Millipore	Cat#AG325
**Glycine** (used at 10 µM)	Bio-Rad	Cat#161-0718
**Glutamate** (used at 100 µM)	Ascent	Cat# Asc-049
**Ifenprodil** (used at 10 µM)	Tocris	Cat#0545
**Ketamine** (used at 500 µM)	Sigma-Aldrich	Cat#K2753
**Lactacystin** (used at 20 µM)	Calbiochem	Cat#426102
**Laminin** (used at 4 µg/ml)	Sigma-Aldrich	Cat#L2020; CAS: 114956-81-9
**Memantine** (used at 10 µM)	Ascent Scientific	Cat#Asc 249
**MTT** (used at 0.5 mg/ml)	Sigma-Aldrich	Cat#M5655; CAS: 298-93-1
**NMDA** (used at 50 or 100 µM as indicated)	Tocris	Cat#0114; CAS: 6384-92-5
**Okadaic acid** (used at 1 µM)	Tocris	Cat#1136; CAS: 78111-17-8
**PhosSTOP** phosphatases inhibitor cocktail tablets	Roche	Cat#04 906 837 001
**Poly-L-Lysine** (used at 100 µg/ml)	Sigma-Aldrich	Cat#P1524; CAS: 25988-63-0
**Prolong** Diamond antifade reagent	Molecular Probes	Cat#P36970
Complete **protease inhibitor** cocktail tablets	Roche	Cat#11 697 498 001
Recombinant **Protein A Sepharose**	Sigma-Aldrich	Cat#11719408001
**Rose Bengal** (used at 20 mg/kg)	Sigma-Aldrich	Cat#R3877; CAS: 632-69-9

**TTC** (used at 2%)	Sigma-Aldrich	Cat#T8877; CAS: 298-96-4
**Cell-penetrating peptides**		
**TMyc** (*YGRKKRRQRRR*AEEQKLISEEDLLR)	GenScript	N/A
**MTMyc** (Ac-*YGRKKRRQRRR*AEEQKLISEEDLLR-NH_2)	GenScript	N/A
**Bio-TMyc** (Biotin-Ahx-*YGRKKRRQRRR*AEEQKLISEEDLLR-NH2)	GenScript	N/A
**$TP95_{29}$** (*YGRKKRRQRRR*LPNQANSPPVIVNT)	GenScript	N/A
**$TP95_{259}$** (*YGRKKRRQRRR*DITTSYSQHLDNEISHSSYLGTDYPT)	GenScript	N/A
**$TP94_{414}$** (*YGRKKRRQRRR*SSLGSGTASLRSNP)	GenScript	N/A
**$MTP95_{414}$** (Ac-*YGRKKRRQRRR*SSLGSGTASLRSNP-NH_2)	GenScript	N/A
**Bio-$TP95_{414}$** Biotin-Ahx-*YGRKKRRQRRR*SSLGSGTASLRSNP-NH_2)	GenScript	N/A
**Primer sequences for quantitative real-time PCR**		
PSD-95: CGTCCTAAGCGGGAATATGA	Sigma-Aldrich	N/A
PSD-95: GTGTGCCTGGATGTCCTTCT	Sigma-Aldrich	N/A
rRNA 18S: CCAGTAAGTGCGGGTCATAAGC	Sigma-Aldrich	N/A
rRNA 18S: CCTCACTAAACCATCCAATCGG	Sigma-Aldrich	N/A
**Critical Commercial Assays**		
BCA Protein Assay Kit	Thermo Fisher	Cat# 23225
Clarity Western ECL Blotting Substrate	BioRad	Cat# 1705060
Kit High Capacity cDNA Reverse Transcription	Applied Biosystems	Cat# 4368814
Lipofectamine 2000	Life Technologies	Cat#11668019
RNeasy Mini Kit	Quiagen	N/A
**Experimental Models: Organisms/Strains**		
Balb/c inbred mice (Balb/cOlaHsd)	Harlan Laboratories	N/A
HEK293T	ATCC	CLS Cat# 300192 /p777_HEK293, RRID:CVCL_0045
Wistar Rat embryos (E18)	In site facility	N/A
**Recombinant DNA**		
pNice-PSD-95-YFP	Dr. P. Scheiffele (Biozentrum, Basel, Suiza)	N/A
**Software and Algorithms**		
CaMPDB (Calpain cleavage prediction using multiple kernel learning; prediction models: MKL, PSSM, SVM linear kernel and SVM RBF kernel)	(DuVerle et al., 2011); http://calpain.org/predict.rb?cls=substrate	RRID:SCR_011976
GeneSilico Metadisorder service	(Kozlowski and	N/A

	Bujnicki, 2012); http://genesilico.pl/metadisorder/	
GPS-Calpain Cleavage Detector	(Liu et al., 2011); http://ccd.biocuckoo.org/	RRID:SCR_000202
ImageJ	https://imagej.net/	RRID:SCR_003070
SitePrediction (predict cleavage site of proteases)	(Verspurten et al., 2009); http://www.dmbr.ugent.be/prx/bioit2-public/SitePrediction/	N/A
Statistical Package for Social Science (SPSS v.18)	IBM (https://www.ibm.com)	RRID:SCR_002865
**Other**		
Protran Western blotting nitrocellulose membrane	GE Healthcare	Cat#GE10600002; CAS: 9004-70-0
PVDF membrane	Pall	BSP0861
Minimum Essential Medium	Life Technologies	Cat#21090-022

Figures and figure legends

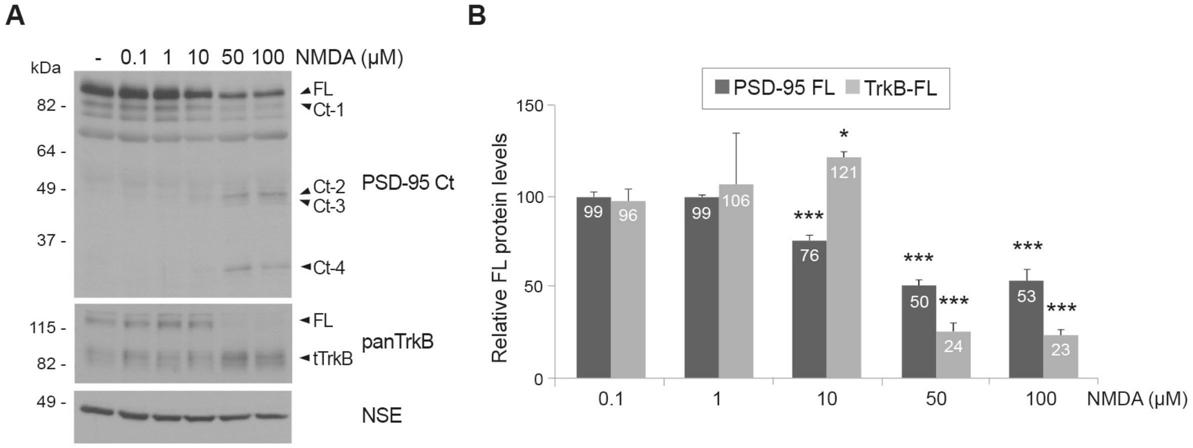

Figure S1. Excitotoxicity-induced PSD-95 downregulation is parallel to that of TrkB-FL. (A) Dose-dependent regulation of PSD-95 and TrkB. Immunoblot analysis of cortical cultures treated with different concentrations of NMDA (0.1-100 µM) for 6 h or left untreated using PSD-95 Ct or panTrkB. Arrows point to PSD-95 FL and TrkB-FL proteins as well as the main PSD-95 C-terminal fragments (Ct-1 to Ct-4) and truncated TrkB forms (TrkB-T1 and calpain truncated TrkB-FL, collectively named tTrkB). A representative experiment is shown. (B) Quantitation of PSD-95 FL and TrkB-FL downregulation. Protein levels were normalized to NSE and expressed relative to those found in the untreated cultures. Means ± SEM are represented (n = 4) and analysis was performed by a generalized linear model followed by a LSD *post-hoc* test. *$p < 0.05$ and ***$p < 0.001$.

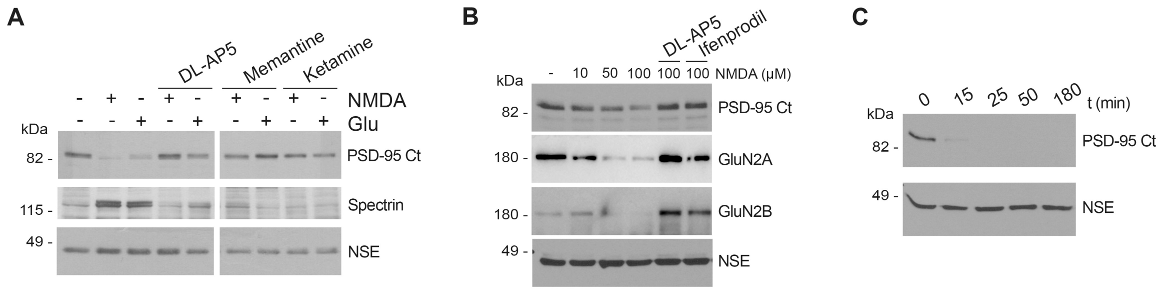

Figure S2. Chronic overactivation of GluN2B-containing NMDARs and acute NMDA treatment induce PSD-95 downregulation. (A) Effect of different NMDAR agonists and antagonists in PSD-95 downregulation induced by excitotoxicity. Primary cortical cultures were treated with NMDA (100 µM) or glutamate (100 µM) together with their co-agonist glycine (10 µM) for 6 h, with or without previous addition of NMDAR antagonists DL-AP5 (200 µM), memantine (10 µM), or ketamine (500 µM). (B) Effect of ifenprodil in PSD-95 downregulation induced by excitotoxicity. Cultures were treated with different NMDA concentrations (10, 50 or 100 µM) for 4 h with or without previous addition of antagonists DL-AP5 (200 µM) or ifenprodil (10 µM), a selective inhibitor of NMDAR-GluN2B subunits. Levels of PSD-95 and NMDAR-subunits GluN2A and GluN2B were established by WB analysis. (C) Analysis of PSD-95 regulation induced by acute excitotoxic damage. Cells were incubated with NMDA (100 µM) as before for different times (15, 25, 50 or 180 min) or left untreated. After agonist removal, cultures proceeded to complete 24 h in the presence of DL-AP5 (200 µM) before protein analysis.

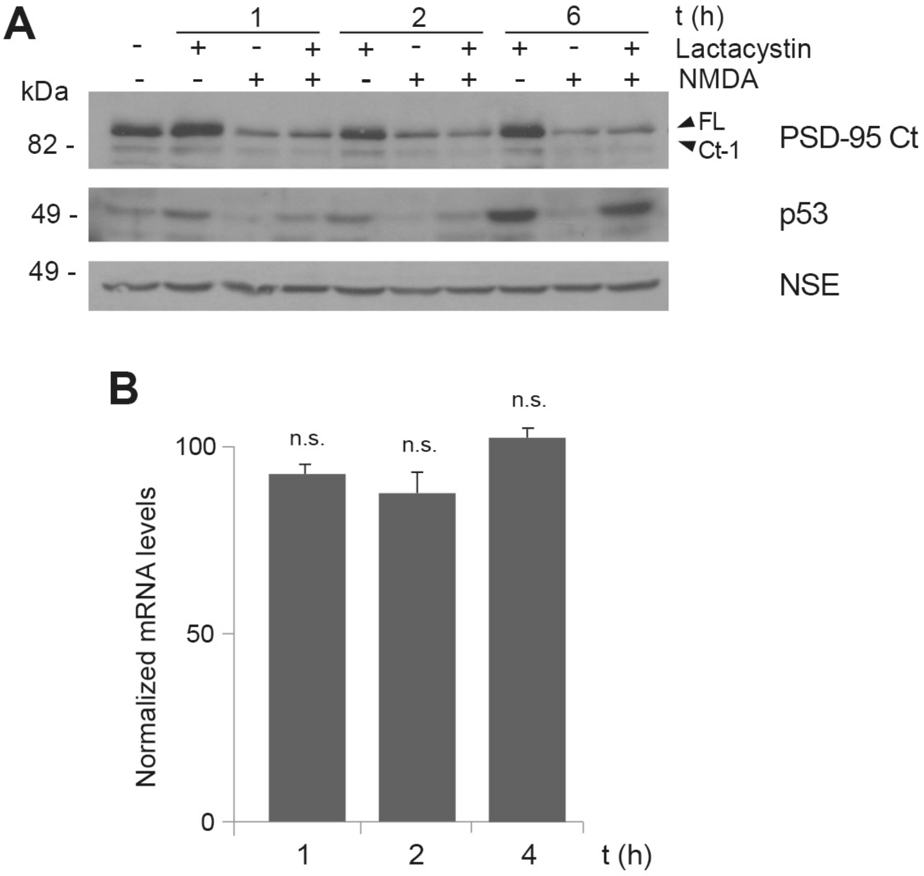

Figure S3. Proteasome activation or transcriptional repression are not involved in PSD-95 downregulation by excitotoxicity. (A) PSD-95 cleavage induced by excitotoxicity is not prevented by proteasome inhibition. Primary cultures were preincubated for 90 min with proteasome inhibitor lactacystin (20 μM) before NMDA treatment as before for 1, 2 or 6 h. The inhibitor was present for the duration of the NMDA treatment. To confirm proteasome inhibition, levels of p53 were analyzed by immunoblot in parallel to those of PSD-95. (B) Transcriptionally-independence of PSD-95 downregulation. Levels of PSD-95 mRNA were established by quantitative RT-PCR of total RNA extracted from cultures treated with NMDA for 1 to 4 h. Results were normalized to those obtained for 18S rRNA, not modified by NMDA treatment. Means ± SEM obtained in excitotoxicity relative to values found in untreated cells are presented (n = 3). Statistical analysis was performed by an ANOVA test followed by a LSD *post-hoc* test. n.s. = non-significant.

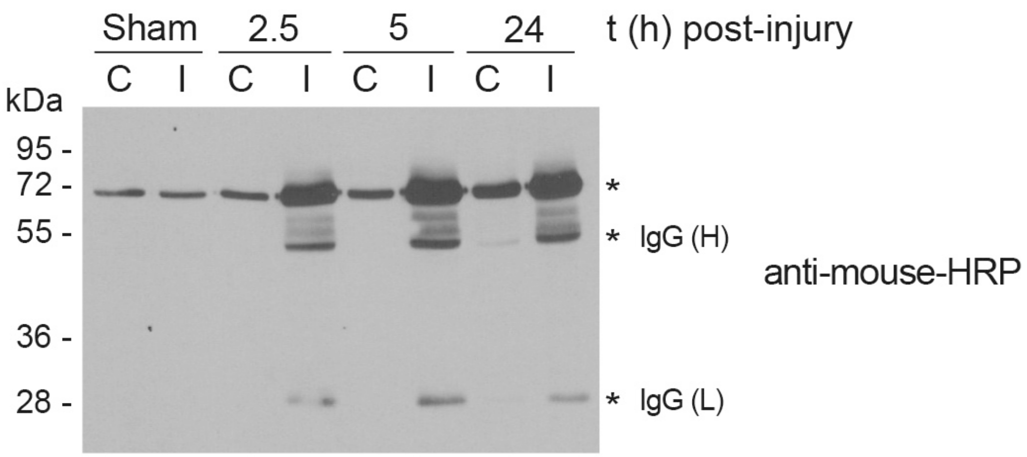

Figure S4. IgG infiltration into the ischemic tissue indicative of BBB breakage takes place at early times after brain damage. Extracts prepared from cortical infarcted regions (I) and equivalent areas of the contralateral hemisphere (C) were compared in mice subjected to ischemia and sacrificed at different times after damage or sham-operated animals. Immunoblot analysis was performed with anti-mouse IgG antibodies. Asterisks mark the major proteins found in the ischemic cortex early after damage induction.

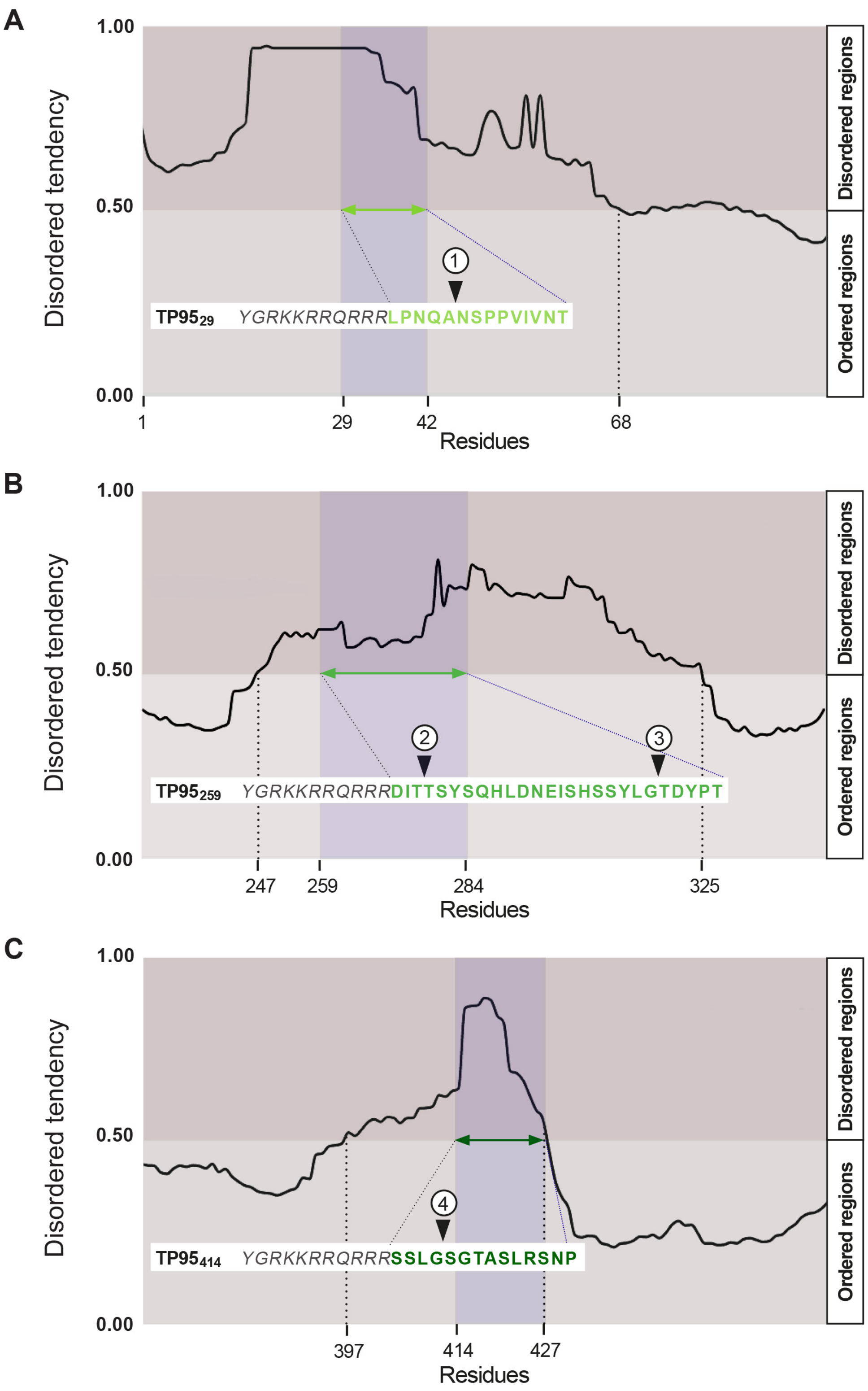

Figure S5. Selected PSD-95 sequences included in peptides TP95$_{29}$, TP95$_{259}$ and TP95$_{414}$ are part of intrinsically disordered regions (IDRs). Detail of PSD-95 IDRs predicted using the online GeneSilico Metadisorder service in relationship to the sequences selected for inclusion in the designed Tat-derived CPPs (highlighted in purple). Residues whose disorder probability is over 0.5 are considered as disordered. The four cleavage sequences established by Edman sequencing are indicated by arrowheads.

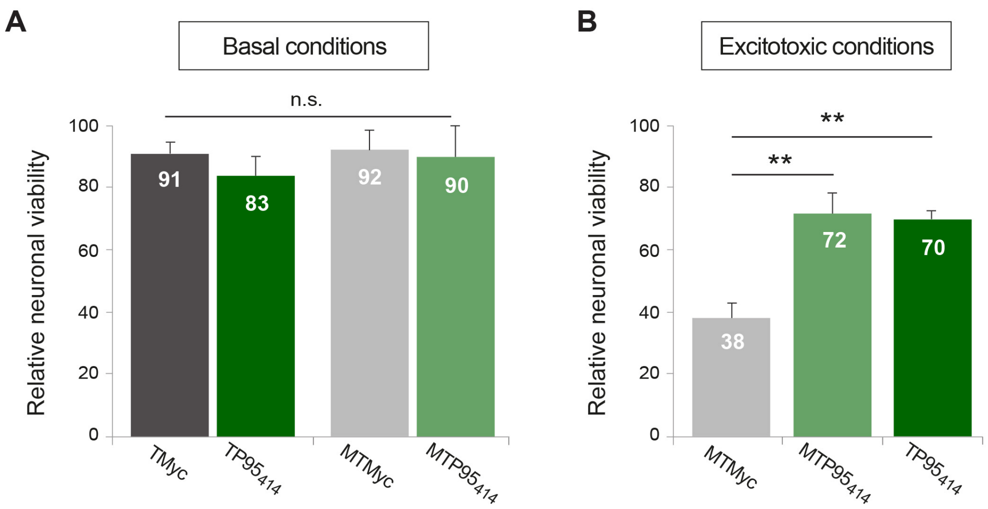

Figure S6. Validation of peptide MTP95$_{414}$ in basal and excitotoxic conditions. (A) Effect of MTMyc and MTP95$_{414}$ on neuronal survival in basal conditions. Primary cultures were incubated with MTMyc and MTP95$_{414}$ (25 µM), or their unmodified versions, for 5 h. Specific neuronal viability was established and expressed relative to values in cultures with no peptide. Means ± SEM are represented (n = 4-5) and statistical analysis was performed by ANOVA test. n.s. = non-significant. (B) Effect of MTMyc and MTP95$_{414}$ on neuronal survival in excitotoxic conditions. Cultures were preincubated for 1 h with MTMyc, MTP95$_{414}$ and TP95$_{414}$ (25 µM) and then treated with NMDA (50 µM) for 2 h. Mean ± SEM values were represented relative to those of neurons incubated with the same peptide but no NMDA (n = 3). Data were analyzed by ANOVA test followed by LSD post-hoc test. **p = < 0.01.
